# Fibroblastic reticular cells mitigate acute GvHD via MHCII-dependent maintenance of regulatory T cells

**DOI:** 10.1172/jci.insight.154250

**Published:** 2022-11-22

**Authors:** Haroon Shaikh, Joern Pezoldt, Zeinab Mokhtari, Juan Gamboa Vargas, Duc-Dung Le, Josefina Peña Mosca, Estibaliz Arellano Viera, Michael A.G. Kern, Caroline Graf, Niklas Beyersdorf, Manfred B. Lutz, Angela Riedel, Maike Büttner-Herold, Alma Zernecke, Hermann Einsele, Antoine-Emmanuel Saliba, Burkhard Ludewig, Jochen Huehn, Andreas Beilhack

**Affiliations:** 1 Interdisciplinary Center for Clinical Research (IZKF), Experimental Stem Cell Transplantation Laboratory, and; 2 Department of Internal Medicine II, Würzburg University Hospital, Würzburg, Germany.; 3 Graduate School of Life Sciences, Würzburg University, Würzburg, Germany.; 4 Laboratory of Systems Biology and Genetics, Institute of Bioengineering, School of Life Sciences, Ecole Polytechnique Fédérale de Lausanne (EPFL), Lausanne, Switzerland.; 5 Department of Experimental Immunology, Helmholtz Centre for Infection Research, Braunschweig, Germany.; 6 Institute for Virology and Immunobiology, Würzburg University, Würzburg, Germany.; 7 Mildred Scheel Early Career Centre, University Hospital of Würzburg, Würzburg, Germany.; 8 Department of Nephropathology, Friedrich-Alexander-Universität Erlangen-Nürnberg, Erlangen, Germany.; 9 Institute of Experimental Biomedicine, University Hospital Würzburg, Würzburg, Germany.; 10 Helmholtz Institute for RNA-based Infection Research (HIRI), Helmholtz-Center for Infection (HZI), Würzburg, Germany.; 11 Institute of Immunobiology, Kantonsspital St. Gallen, St. Gallen, Switzerland.; 12 Institute of Experimental Immunology, University of Zurich, Zurich, Switzerland.; 13 Cluster of Excellence RESIST (EXC 2155), Hannover Medical School, Hannover, Germany.

**Keywords:** Hematology, Transplantation, Antigen-presenting cells, MHC class 2, Stem cell transplantation

## Abstract

Acute graft versus host disease (aGvHD) is a life-threatening complication of allogeneic hematopoietic cell transplantation (allo-HCT) inflicted by alloreactive T cells primed in secondary lymphoid organs (SLOs) and subsequent damage to aGvHD target tissues. In recent years, Treg transfer and/or expansion has emerged as a promising therapy to modulate aGvHD. However, cellular niches essential for fostering Tregs to prevent aGvHD have not been explored. Here, we tested whether and to what extent MHC class II (MHCII) expressed on Ccl19^+^ fibroblastic reticular cells (FRCs) shape the donor CD4^+^ T cell response during aGvHD. Animals lacking MHCII expression on *Ccl19*-Cre–expressing FRCs (MHCII^ΔCcl19^) showed aberrant CD4^+^ T cell activation in the effector phase, resulting in exacerbated aGvHD that was associated with significantly reduced expansion of Foxp3^+^ Tregs and invariant NK T (iNKT) cells. Skewed Treg maintenance in MHCII^ΔCcl19^ mice resulted in loss of protection from aGvHD provided by adoptively transferred donor Tregs. In contrast, although FRCs upregulated costimulatory surface receptors, and although they degraded and processed exogenous antigens after myeloablative irradiation, FRCs were dispensable to activate alloreactive CD4^+^ T cells in 2 mouse models of aGvHD. In summary, these data reveal an immunoprotective, MHCII-mediated function of FRC niches in secondary lymphoid organs (SLOs) after allo-HCT and highlight a framework of cellular and molecular interactions that regulate CD4^+^ T cell alloimmunity.

## Graphical abstract

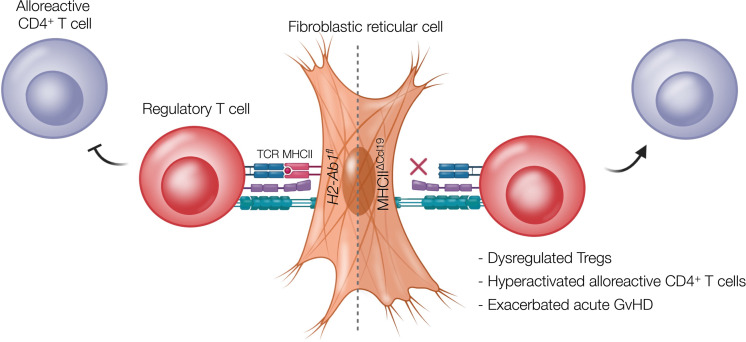

## Introduction

Hematopoietic cell transplantation (HCT) is the only reliable curative therapy for various hematological malignancies and genetic hematological disorders (1–3). Nevertheless, acute graft versus host disease (aGvHD) remains the leading cause of morbidity and mortality (20%) of transplant-related complications following allogeneic HCT (allo-HCT) (4, 5). FoxP3-expressing Tregs are pivotal for the regulation of self-reactive T cells and autoimmunity (6) and have been clinically shown to prevent aGvHD without impairing the graft versus leukemia (GvL) effect (7, 8). However, the cellular niches that are essentially required to foster Treg homeostasis and function after allo-HCT have not been fully addressed.

Secondary lymphoid organs (SLOs) serve as initiation sites of aGvHD (9). Within SLOs, nonhematopoietic lymph node stromal cells (LNSCs) provide the infrastructure to maintain immune homeostasis and facilitate rapid and effective immune responses (10–12). Fibroblastic reticular cells (FRCs) are immunologically specialized fibroblasts of mesenchymal origin and comprise 20%–50% of the nonhematopoietic compartment of SLOs (13, 14) forming the conduit network, maintaining the reticular network tension and LN expansion (15). Furthermore, FRCs are a major source of naive T/B cell survival factors (16, 17) and secrete the homeostatic chemokines CCL19 and CCL21 to mediate the recruitment of CCR7-expressing naive T cells (18). Activated FRCs play a crucial role in both limiting (19–23) and promoting (24, 25) T cell responses. FRCs reportedly express MHC class II (MHCII) and directly acquire self-peptide/MHCII complexes from DCs to present to and tolerize antigen-specific CD4^+^ T cells (26, 27). Furthermore, FRCs indirectly restrain aberrant CD4^+^ T cell activation via MHCII presentation of self-antigen to induce proliferation of Tregs (28, 29). GvHD causes irreversible damage to FRC populations that result in impaired capacity to display peripheral tissue–restricted antigen in the SLOs, leading to autoimmunity (30–32). On the contrary, certain FRC subsets provide critical Delta-like 1/4 (DLL1/4) Notch ligands for the in vivo priming of alloreactive T cells (33, 34), suggesting that FRCs play a critical role in initiating alloreactive T cell responses leading to aGvHD.

Naive allogeneic T cells primarily home to SLOs within hours after allo-HCT (35). Thus, FRC topology within SLOs and their functions in modulating immunity make them interesting candidates to investigate their potential role in modulating alloimmune responses. The activation of alloreactive T cells primarily occurs in the SLOs, including spleen, LNs and Peyer’s patches (PPs) (9, 35). In recent years, it has been demonstrated that CD4^+^ T cell–mediated alloresponses can occur in the absence of hematopoietic antigen presenting cells (APCs) as a source of allo-antigens (36–38), implying that our current understanding remains incomplete regarding which nonhematopoietic APCs drive and which regulate alloreactive T cells during aGvHD initiation and effector phases.

Here, we used a combination of mouse models with cell type–specific antigen expression and loss of function to interrogate the in vivo spatial and temporal requirements of MHCII presentation by FRCs during the initiation and effector phases of aGvHD. We found that, after myeloablative irradiation, FRCs upregulated costimulatory receptors and degraded and processed exogenous antigen using the MHCII machinery. Surprisingly, lack of MHCII on FRCs that are lineage traced with the *Ccl19*-Cre transgene had no influence on alloreactive CD4^+^ T cell activation and proliferation in the initiation phase of aGvHD. However, ablation of MHCII on Ccl19^+^ FRCs dysregulated FoxP3^+^ Tregs, resulting in the hyperactivation of CD4^+^ conventional T cells (Tcon), which exacerbated aGvHD.

## Results

### MHCII-related genes are distinctly expressed on LN SC subsets.

**Figure 1:**
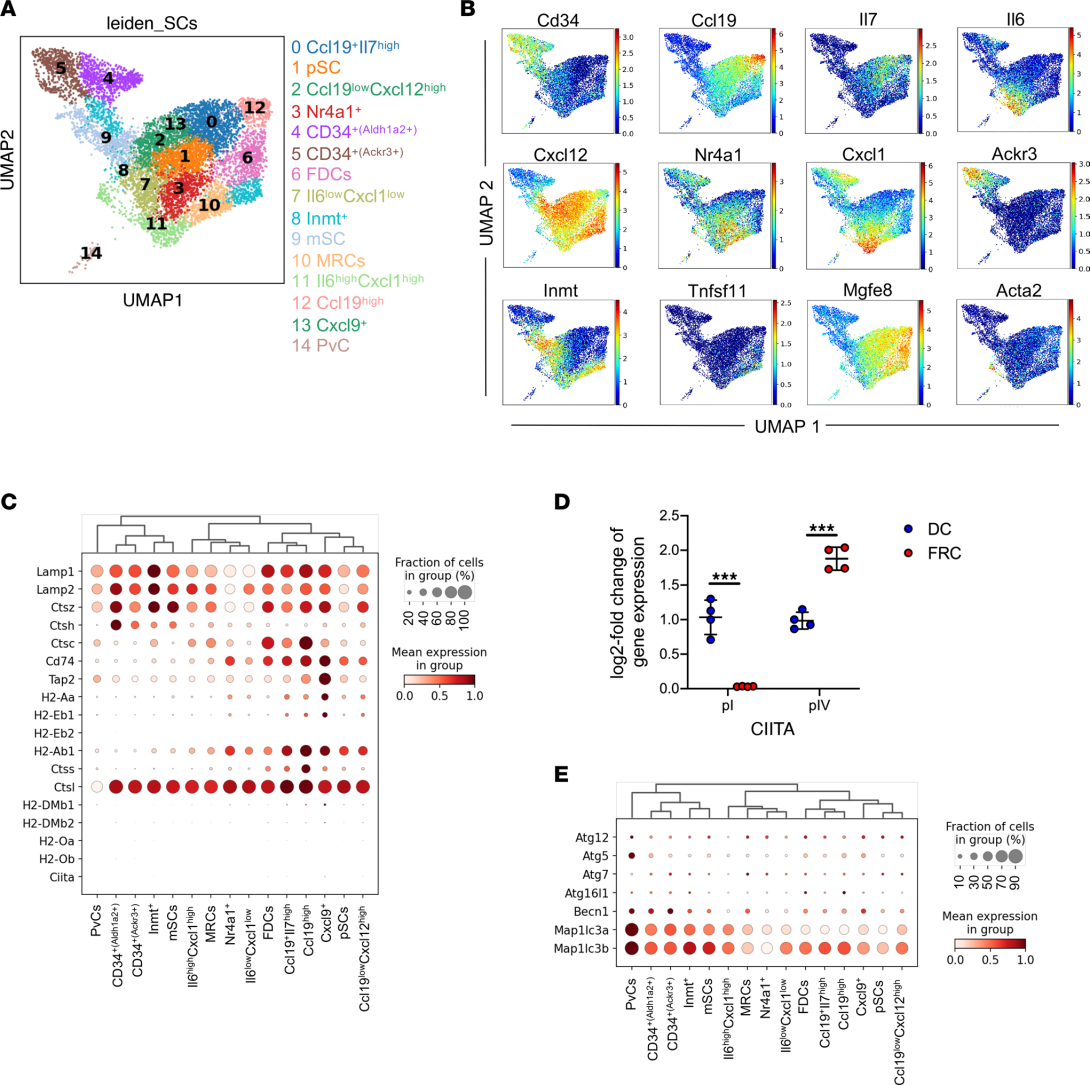
scRNA-Seq reveals differential expression of MHCII-mediated antigen presentation in the SCs subset of LNs. Single-cell suspension from mLNs and pLNs were sorted for CD45^–^CD24^–^ cells and subjected to scRNA-Seq. Endothelial cells were identified as *Pecam*^+^ and were excluded from further downstream analysis. Data shown are pooled from 2 mLNs (sample 1, 2,247 cells; sample 2, 1,339 cells) and 2 pLNs (sample 1, 2,935 cells; sample 2, 2,757 cells) data sets. (**A**) UMAP plot of merged mLNs SCs and pLNs SCs showing cluster segregation. (**B**) Expression of subset defining DEGs across SCs on UMAP plot. (**C**) Heatmap of expression of genes involved in expression of MHCII-mediated antigen presentation genes on 15 identified clusters of SCs. (**D**) Expression of CIITA pI and pIV on DCs and FRCs evaluated by qPCR. Data are from 1 experiment, and 1 data point represents 1 mouse. Two-tailed unpaired Student’s *t* test was used; data are shown as mean± SD. ***P < 0.001. (**E**) Expression of autophagy on 15 identified clusters of SCs. mLNs, mesenteric lymph nodes; pLNs, peripheral lymph nodes, pLNs; stromal cells, SCs; DEGs, differentially expressed gene; UMAP, Uniform manifold approximation and projection for dimension reduction.

LN reticular cells are a heterogeneous population of cells and, based on their niches in the LNs, display distinct characteristics, function, and gene expression. First, we addressed whether FRCs can serve as potential APCs to modulate allogeneic CD4^+^ T cells. To evaluate the expression of genes involved in MHCII-mediated antigen processing and presentation, we performed an in-depth single-cell RNA-Seq (scRNA-Seq) in silico analysis of the entire CD45^–^CD24^–^ population from mesenteric and peripheral LNs of steady-state adult BALB/c mice (39). Endothelial cells were excluded from the analysis based on the expression of *Pecam1* (not shown). An unbiased uniform manifold approximation and projection (UMAP) for dimension reduction clustering of SC subsets within LNs revealed fifteen transcriptional unique clusters (clusters 0–14) harboring unique functional profiles as identified by differentially expressed genes (DEGs) (Figure 1, A and B). We could recapitulate and identify SC subsets as previously reported (39, 40). Subsets that have been previously described with high but not exclusive expression of *Nr4a1* and *Inmt* were termed as Nr4a1^+^ and Inmt^+^ SCs, respectively (39, 40). Clusters proliferating and metabolically active were termed as proliferating SCs (pSC) and metabolically active SCs (mSC), respectively, on their DEGs profile. Whereas marginal reticular cells (MRCs) were assigned by their distinguished expression of *Enpp2*, *Tnfsf11*, and follicular DCs (FDCs) by the expression of *Mfge8* (40) (Supplemental Figure 1A; supplemental material available online with this article; https://doi.org/10.1172/jci.insight.154250DS1).

Furthermore, we identified 4 clusters with differential expression of *CD34*; 2 of these clusters were classified as CD34^+(Ackr3+)^ and CD34^+(Aldh1a2+)^. Conversely, *Ccl19* — a known chemokine that attracts naive T cells and DCs — was observed to be expressed at different levels on 12 of 15 cluster, except perivascular cell (PvCs), CD34^+(Ackr3+)^, and CD34^+(Aldh1a2+)^. These *Ccl19*-expressing SCs were characterized on their expression profile of previously known gene signatures of *Il6*, *Il7*, *Cxcl1*, and *Cxcl9* (39, 40), as well as the potentially novel DEG *Cxcl12*, which is identified in our analysis (Supplemental Figure 1A).

Having identified SC subsets, we next evaluated the gene expression profile involved in MHCII-mediated antigen processing and presentation. Lysosomal membrane markers *Lamp1* and *Lamp2* were expressed by all subset of SCs with different expression levels. Genes involved in lysosomal processing: cathepsin Z (*ctsz*) was expressed in all subsets of SCs at different levels, whereas cathepsin H (*ctsh*) was highly expressed in CD34^+(Aldh1a2+)^ SCs and cathepsin C (*ctsc*) in Ccl19^hi^ SCs (FRCs). Invariant chain (CD74 or li) is processed by either cathepsin S (*ctss*) or cathepsin L (*ctsl*) (41–43). In our analysis, we observed *ctsl* to be expressed by all SC subsets, whereas *ctss* was exclusively expressed by Ccl19^hi^ cells comprising FRCs. Nevertheless, expression of genes involved in MHCII stability and antigen presentation, like *Cd74*, *Tap2*, *H2-Aa*, *H2-Eb1*, *H2-Eb2*, and *H2-Ab1*, were strikingly higher in SCs subsets that coexpressed Ccl19 (i.e., clusters Ccl19^+^Il7^hi^, Ccl19^hi^, Cxcl9^+^, pSCs, and Ccl19^lo^Cxcl12^hi^) and define FRC subpopulations (Figure 1C). Expression of class II transactivator (CIITA), the master regulator of MHCII expression was not detectable in our scRNA data set; however, gene expression analysis revealed that FRCs exclusively express promoter IV (pIV) of CIITA (Figure 1D), consistent with previous reports (26).

In addition to conventional antigen processing, intracellular and extracellular antigens are also processed via MHCII presentation by macroauthophagy. We observed the enrichment of autophagosome recruitment genes in PvCs, CD34^+(Ackr3+)^, CD34^+(Aldh1a2+)^, and Inmt^+^ clusters (Figure 1E). Moreover, at steady state, all SC subsets had little to no expression of costimulatory and coinhibitory molecules (Supplemental Figure 1, B and C).

These scRNA-Seq data suggest that Ccl19^+^ FRCs may execute critical APC functions and could play a major role in modulating allogeneic CD4^+^ T cells.

### Activation of FRCs after irradiation.

**Figure 2:**
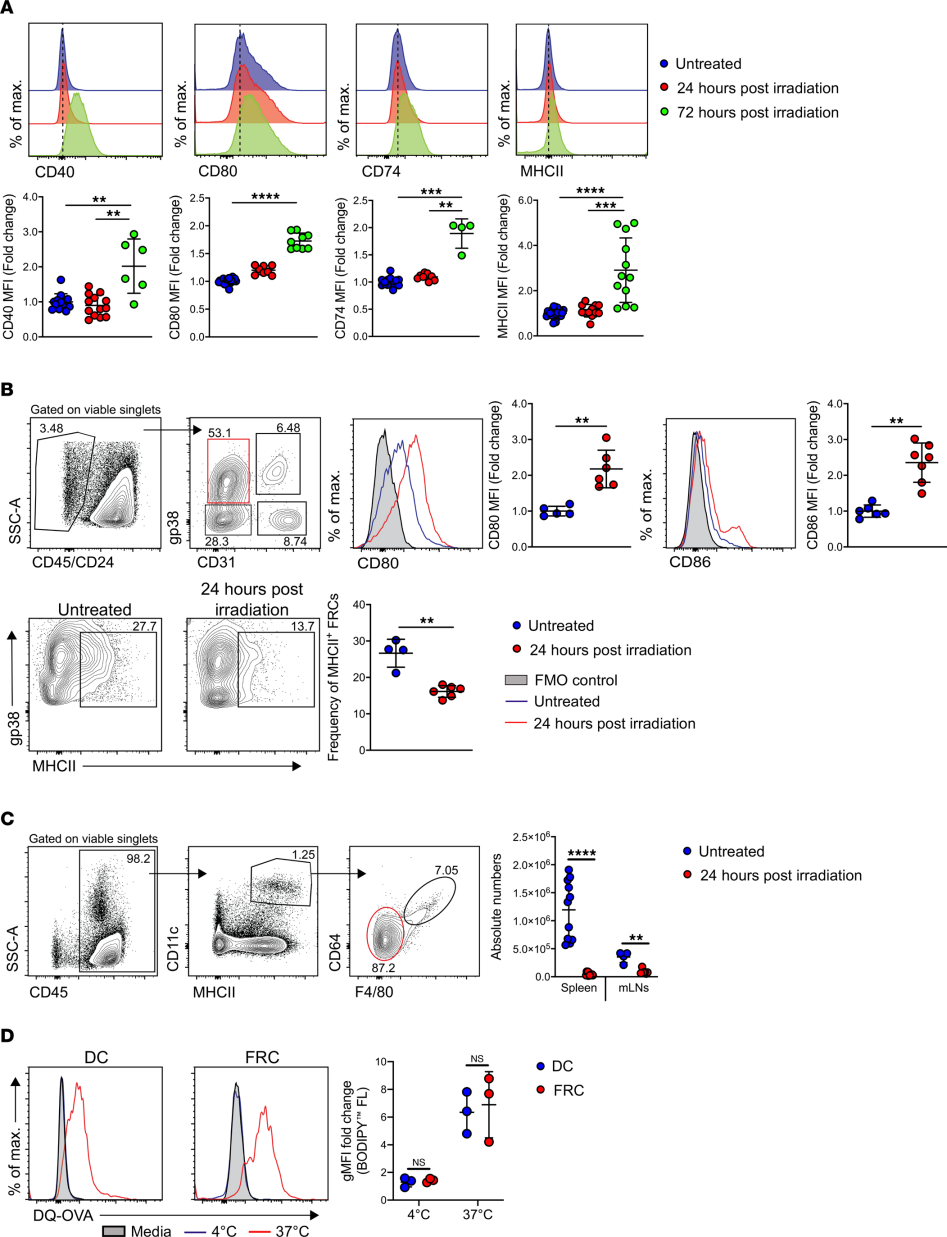
Fibroblastic reticular cells upregulate costimulatory molecules after irradiation. (**A**) Expression of costimulatory receptors as normalized mean fluorescent intensity (MFI) on untreated immortalized FRC cell line, 24 hours and 72 hours after irradiation (30 Gy). (**B**) Gating for FRCs (gp38^+^CD31^–^) and expression of CD80 and CD86 as normalized MFI on FRCs and frequency of MHCII^+^ FRCs at steady state and 24 hours after lethal irradiation (9 Gy) in C57BL/6 mice. (**C**) Gating and absolute numbers of DCs (CD45^+^CD11c^+^MHCII^+^CD64^–^F4/80^–^) at steady state and 24 hours after myeloablatively irradiation (9 Gy) from spleen and mesenteric lymph nodes in C57BL/6 mouse. (**D**) Splenic DCs and lymph node FRCs sorted by FACS from C57BL/6 mouse incubated with DQ-OVA for 3 hours at 4°C and 37°C, followed by analysis of processed DQ-OVA (blue laser 488 nm, BL-1) normalized to media. Data pooled from 2 experiments, with 1 data point representing 1 mouse. Unpaired nonparametric Mann-Whitney *U* test was used; data are shown as mean± SD. **P *<* 0.01, ***P *<* 0.001, and ****P *<* 0.0001.

Professional hematopoietic APCs upregulate MHCII and costimulatory receptors (CD40, CD80, and CD86) under inflammatory conditions triggered by infection, injury, and/or stress (44, 45). Therefore, we assessed next whether host conditioning such as irradiation and/or chemotherapy prior to allo-HCT could trigger FRCs to acquire an APC phenotype. Hence, we irradiated a mouse immortalized FRC (iFRCs) cell line (15) that had been isolated by FACS of CD45^–^CD31^–^ and podoplanin^+^ (gp38) population from peripheral LNs of C57BL/6 mice, and we examined the expression of CD40 (TNFRSF5 [tumor necrosis factor receptor superfamily member 5]), CD80 (B7-1), CD74 (invariant chain), and MHCII (I-Ab) (28) 24 and 72 hours after irradiation compared with nonirradiated steady-state iFRCs. iFRCs upregulated CD40 and CD80 costimulatory receptors within 24 hours of irradiation and further enhanced expression by 72 hours. Likewise, iFRCs upregulated crucial surface molecules for antigen presentation, such as MHCII and its invariant chain CD74 at 24 and 72 hours after irradiation (Figure 2A). To recapitulate these findings in vivo, we myeloablatively irradiated C57BL/6 mice. Flow cytometry analysis revealed that gp38^+^ FRCs upregulated costimulatory molecules (CD80 and CD86) within 24 hours after conditioning (Figure 2B). However, frequency of MHCII-expressing FRCs was reduced in vivo (Figure 2B). Others have shown that FRCs acquire MHCII from DCs via endocytosis (26). Following irradiation, DC numbers dramatically decreased in spleens and mesenteric LNs (mLNs) (Figure 2C), suggesting that the loss of MHCII on FRCs after conditioning may occur due to rapid depletion of radiosensitive DCs in the lymphoid organs. Furthermore, treatment with a chemotherapeutic agent, Gemcitabine, resulted in the upregulation of MHCII on FRCs, whereas the expression of CD80 and CD86 remained unaltered (Supplemental Figure 2A). Next, we assessed whether FRCs degrade and process exogenous antigen under homeostatic, noninflammatory conditions. Culturing FACS-sorted LN-derived FRCs at 37°C with DQ-OVA (a self-quenched conjugate of OVA that exhibits bright green fluorescence upon *ctsl* and pH-dependent degradation) resulted in processing of DQ-OVA at similar levels as observed in splenic DCs (CD45^+^CD11c^+^MHCII^+^CD64^–^F4/80^–^) sorted by FACS that served as hematopoietic professional APC control (Figure 2D). Furthermore, FRCs isolated from MHCII^ΔVav1^ mice, in which DCs are devoid of MHCII, were able to process DQ-OVA, indicating that FRC-endogenous MHCII machinery is sufficient to process exogenous antigens in the absence of MHCII transfer from DCs (Supplemental Figure 2B). Similarly, FRCs could also process DQ-OVA 48 hours after allo-HCT (Supplemental Figure 2C). Taken together, these findings suggest that FRCs have the capacity to modulate allogeneic CD4^+^ T cells in an aGvHD inflammatory environment.

### Activation of alloreactive CD4^+^ T cells during aGvHD.

**Figure 3:**
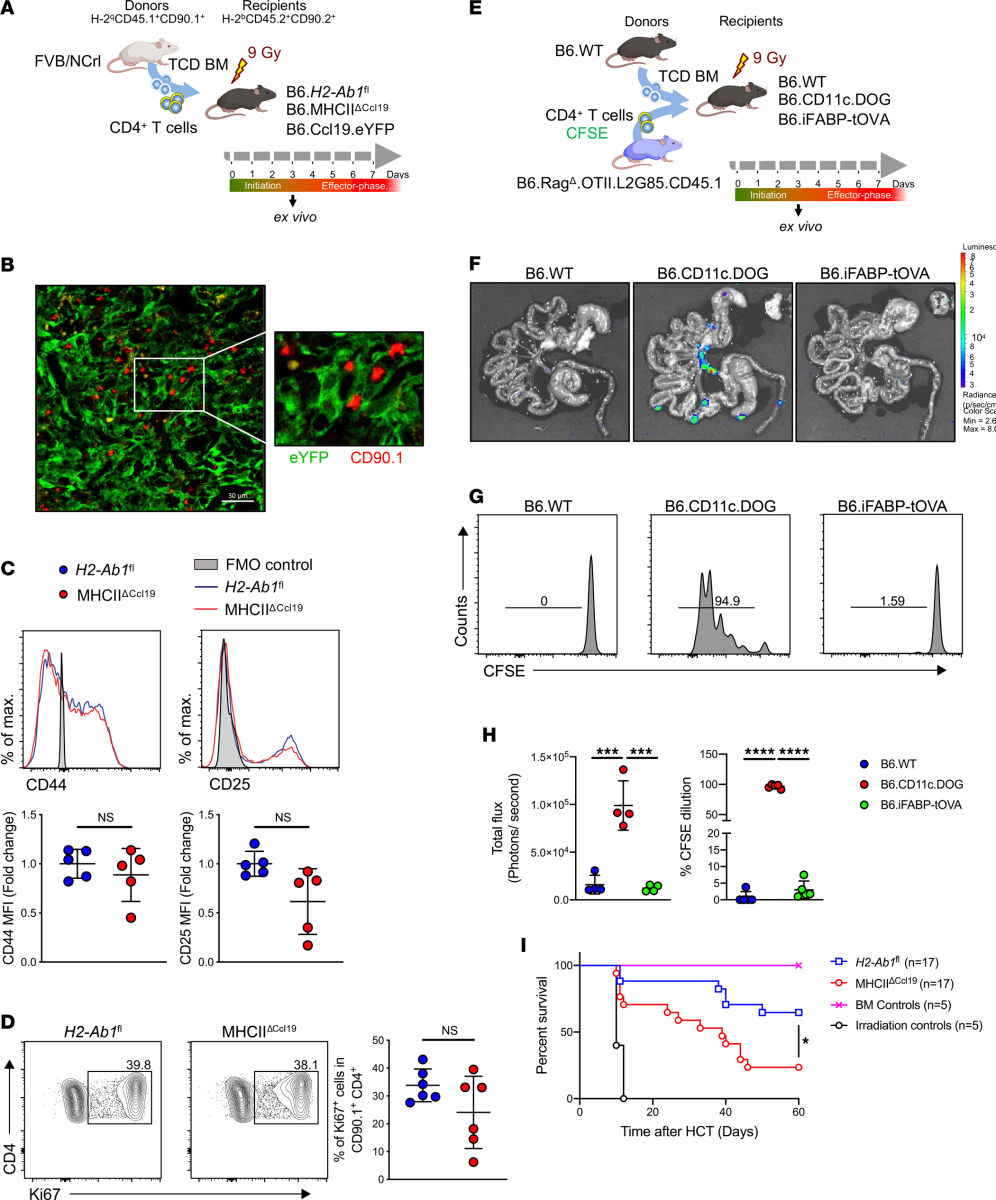
MHCII on FRCs is dispensable for allogeneic CD4^+^ T cell activation during the initiation phase of aGvHD. (**A**) Experimental strategy. B6.*H2-Ab1*^fl^, B6.MHCII^ΔCcl19^, and B6.Ccl19.eYFP recipients were myeloablatively irradiated with 9 Gy and i.v. transplanted with 5 × 10^6^ T cell-depleted (TCD) BM and 6 × 10^5^ CD4^+^ T cells from FVB/N mice and ex vivo analyzed day 3 of allo-HCT. (**B**) Representative microphotographs of donor CD4^+^ T cells stained with CD90.1^+^ along with YFP expressed by SCs in B6.Ccl19-eYFP mice at day 3 of allo-HCT in LNs. (**C**) Normalized MFI of CD44 and CD25. (**D**) Frequency of Ki67^+^ on donor CD4^+^ T cells (CD90.1^+^CD4^+^) at day 3 of allo-HCT in spleen. (**E**) Experimental strategy. C57BL/6 (B6.WT), B6.CD11c.DOG (expressing OVA in myeloid antigen-presenting cells), and B6.iFABP-tOVA recipients (expressing OVA on FRCs and intestinal epithelial cells) were myeloablatively irradiated with 9 Gy and i.v. transplanted with 5 × 10^6^ TCD-BM and 1 × 10^6^ OT-II CD4^+^ T cells from B6.WT and B6.Rag^Δ^.OTII.L2G85.CD45.1 mice, respectively, and analyzed on day 3 of syn-HCT. (**F**) Ex vivo bioluminescence imaging (BLI) micrograph. (**G**) CFSE dilution on adoptively transferred CD4^+^CD45.1^+^ T cells in mLNs. (**H**) Quantification of the adoptively transferred OT-II T cells (CD45.1^+^CD4^+^) BLI signal and CFSE dilution in mLNs on day 3 of syn-HCT. Data were pooled from 2 experiments, with 1 data point representing 1 mouse. Two-way ANOVA with Tukey’s test was used; data are shown as mean± SD. ***P < 0.001 and ****P < 0.0001. (**I**) Survival of myeloablatively irradiated (9 Gy) B6.*H2-Ab1*^fl^ and B6.MHCII^ΔCcl19^ mice transplanted with 5 × 10^6^ TCD-BM and 6 × 10^5^ enriched CD4^+^ T cells from FVB/N mice illustrated in Kaplan-Meier curve.

To further elucidate the role of MHCII expression by Ccl19^+^ FRCs in relation to the modulation of alloreactive CD4^+^ T cells, we deleted MHCII in all Ccl19-Cre–expressing SCs (MHCII^ΔCcl19^), resulting in an effective KO (Supplemental Figure 3, A and B) (46, 47). These mice showed frequencies of conventional CD4^+^ and CD8^+^ Tcon, as well as CD4^+^FoxP3^+^ Tregs comparable with WT *H2-Ab1*^fl^ littermates at steady state (Supplemental Figure 4, A and B). Consequently, we transplanted B6.Ccl19-eYFP, B6.MHCII^ΔCcl19^, and WT *H2-Ab1*^fl^ littermates with allogeneic FVB/N BM and CD4^+^ T cells after myeloablative conditioning (Figure 3A). During the initiation phase of aGvHD (day 3 after allo-HCT), we observed allogeneic CD4^+^ T cells expressing CD90.1, colocalized with eYFP-expressing SCs (Figure 3B and Supplemental Figure 5A). However, we did not observe significant differences in alloreactive donor CD4^+^ T cell activation between MHCII^ΔCcl19^ and WT *H2-Ab1*^fl^ littermate recipient mice as assessed by CD44 and CD25 expression (Figure 3C); furthermore, proliferation (Figure 3D and Supplemental Figure 5B) and effector profiles (Supplemental Figure 5C) were marginally reduced but not significantly altered in the MHCII^ΔCcl19^ mice when compared with MHCII-competent controls in mLNs and spleen (data not shown). Moreover, to further dissect direct antigen presentation by FRCs to CD4^+^ T cells, we employed an OVA transgenic model of intestinal fatty-acid binding protein (iFABP-tOVA) mice (48), in which truncated OVA (tOVA) amino acids 138–386 are expressed on intestinal epithelial cells, as well as ectopically on FRCs (48–50) (Supplemental Figure 5D). When OT-II–specific CD4^+^ T cells from B6.Rag^Δ^.OTII.L2G85.CD45.1 mice were adoptively transferred into myeloablatively irradiated B6.CD11c.DOG mice expressing OVA on CD11c^+^ cells (Figure 3E), they excessively proliferated in the mLNs and PPs within 72 hours. However, OT-II T cells failed to proliferate in the same SLOs of B6.iFABP-tOVA mice (Figure 3, F–H). Furthermore, CD4^+^ OT-II cells failed to infiltrate and cause intestinal injury in B6.iFABP-tOVA mice, whereas CD8^+^ OT-I cells efficiently infiltrated intestinal tissue and proliferated (Supplemental Figure 5, E and F), causing severe disease and mortality (Supplemental Figure 5G).

These data suggest that alloreactive CD4^+^ T cells can be activated to proliferate at least largely independently of MHCII antigen presentation by Ccl19^+^ FRCs during the initiation phase of aGvHD. However, surprisingly, MHCII^ΔCcl19^ mice showed an exacerbated disease phenotype during the effector phase of aGvHD, resulting in significantly worse survival (Figure 3I) and suggesting dysregulation of immune-regulatory mechanisms.

### MHCII expression by FRCs regulates the effector phase of aGvHD.

Downregulation of MHCII on FRCs following host conditioning (Figure 2B) was reversed in the aGvHD effector phase (day 30 of allo-HCT) when FRCs significantly upregulated MHCII, yet MHCII expression levels remained markedly lower compared with steady state (Supplemental Figure 6A). To explore the importance of MHCII on FRCs in the effector phase of aGvHD, we assessed donor CD4^+^ T cell at various time points after allo-HCT. Two weeks after allo-HCT, we observed hyperactivation of donor CD4^+^ T cells as marked by the significantly upregulated expression of CD44 in the spleen and markedly increased in intraepithelial cell (IEL) fraction and lamina propria (LP) donor T cells (Supplemental Figure 6B), whereas killer cell lectin-like receptor subfamily G member 1 (KLRG1), a marker of effector Tregs, was reduced on Tregs in all the organs evaluated and Helios remained unchanged at this time point (Supplemental Figure 6C).

**Figure 4:**
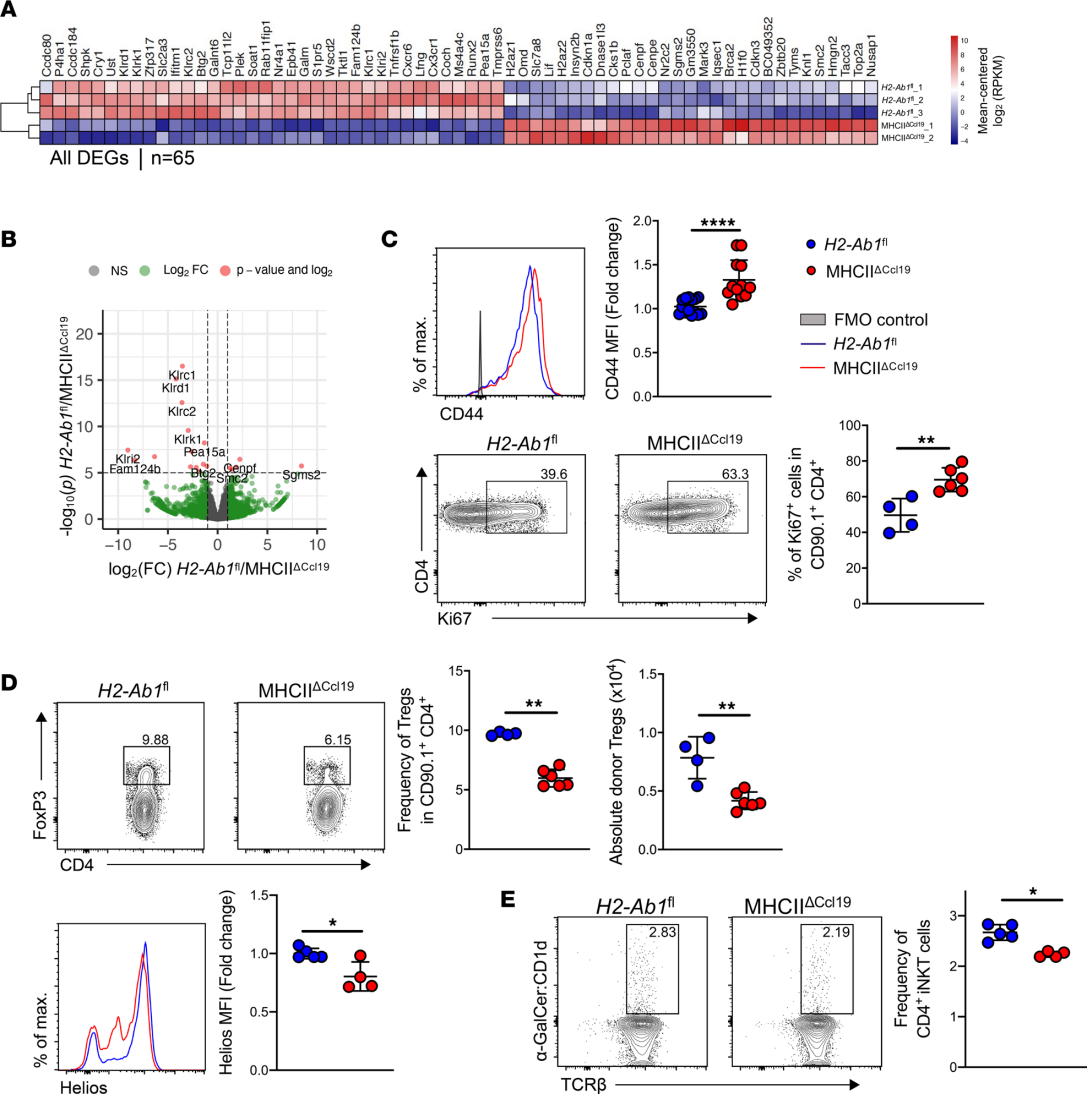
Allogeneic CD4^+^ T cells are hyperactivated in MHCII^ΔCcl19^ mice during the effector phase of aGvHD. (**A**) Heatmap of significant differentially expressed genes (DEGs, log_2_ fold change ≥ 0.75, *q* *≤* 0.05) on allogeneic CD4^+^ T cells in MHCII^ΔCcl19^ versus *H2-Ab1*^fl^ mice in the effector phase of allo-HCT from spleen. (**B**) DEGs in CD4^+^ T cells from MHCII^ΔCcl19^ mice depicted as volcano plot. (**C**) Expression of T cell activation molecule in normalized MFI CD44 and frequency of Ki67^+^ by donor allogeneic CD4^+^ T cells (CD90.1^+^CD4^+^). (**D**) Frequency, absolute numbers of donor allogeneic Tregs (CD90.1^+^CD4^+^FoxP3^+^) in CD90.1^+^CD4^+^ cells and expression of Helios in normalized MFI. (**E**) Frequency of iNKT cells (α-GalCer:CD1d^hi^TCRβ^hi^) in donor allogeneic CD4^+^ T cells (CD90.1^+^CD4^+^) at day +30 of allo-HCT in LNs. Data were pooled from 2 experiments, with 1 data point representing 1 mouse. Unpaired nonparametric Mann-Whitney *U* test was used; data are shown as mean± SD. *P < 0.05, **P < 0.01, and ****P < 0.0001.

RNA-Seq of alloreactive CD4^+^ T cells from spleen at day 30 of allo-HCT revealed significant enrichment of genes involved in mitosis (*Cdkn1a*, *Cks1b*, *Pclaf*, *Dnase1l3*, *Cenpf*, *Cenpe*, and *Cdkn3*), chromatin remodeling (*H2Az1*, *H2Az2*, *Hmgn2*, *H1f0*, *Knl1*, *Smc2*, *Tacc3*, *Topa2*, and *Nusap1*), growth, and cellular differentiation (*Lif*, *Nr2c2*, *Sgms2*, *Iqsec1*, *Brca2*, and *Zbtb20*), suggesting that donor CD4^+^ T cells in MHCII^ΔCcl19^ recipients were in a higher activation state compared with *H2-Ab1*^fl^ recipients (Figure 4, A and B, and Supplemental Figure 6D). In contrast, allogeneic CD4^+^ T cells in MHCII^ΔCcl19^ mice downregulated genes involved in (a) glucose metabolism (*Shpk*, *Ust*, *Slc2a2*, *Galm*, *Tktl2*, *Tnfrsf1b*, *Lfng*, and *Runx2*), suggesting T cells utilizing alternate metabolic pathways support biosynthesis and anti-proliferative proteins (*Btg2*, *S1pr5,* and *Ifitm1*); (b) cell adhesion and extracellular matrix (*Ccdc80*, *P4ha1*, *Galnt6*, and *Tmprss6*); and (c) activity of invariant NK T (iNKT) cells (*Klrd1*, *Klrk1*, *Klrc1*, *Klrc2*, and *Klri2*), suggesting loss of CD4^+^ iNKT cells in MHCII^ΔCcl19^ mice (Figure 4, A and B). At this time point, allogeneic CD4^+^ T cells in the spleen of MHCII^ΔCcl19^ recipients displayed an effector CD44^+^ phenotype, and more CD4^+^ T cells were proliferating (Ki67^+^) when compared with control recipients (Figure 4C). Notably, the frequency, absolute numbers of donor-derived Tregs (CD90.1^+^CD4^+^FoxP3^+^) (Figure 4D, left), and expression of Helios on Tregs (Figure 4D, right), as well as that of iNKT cells (CD90.1^+^CD4^+^α-GalCer:CD1d^hi^TCRβ^hi^) (Figure 4E), were significantly reduced in MHCII^ΔCcl19^ mice after day 30 of allo-HCT.

**Figure 5:**
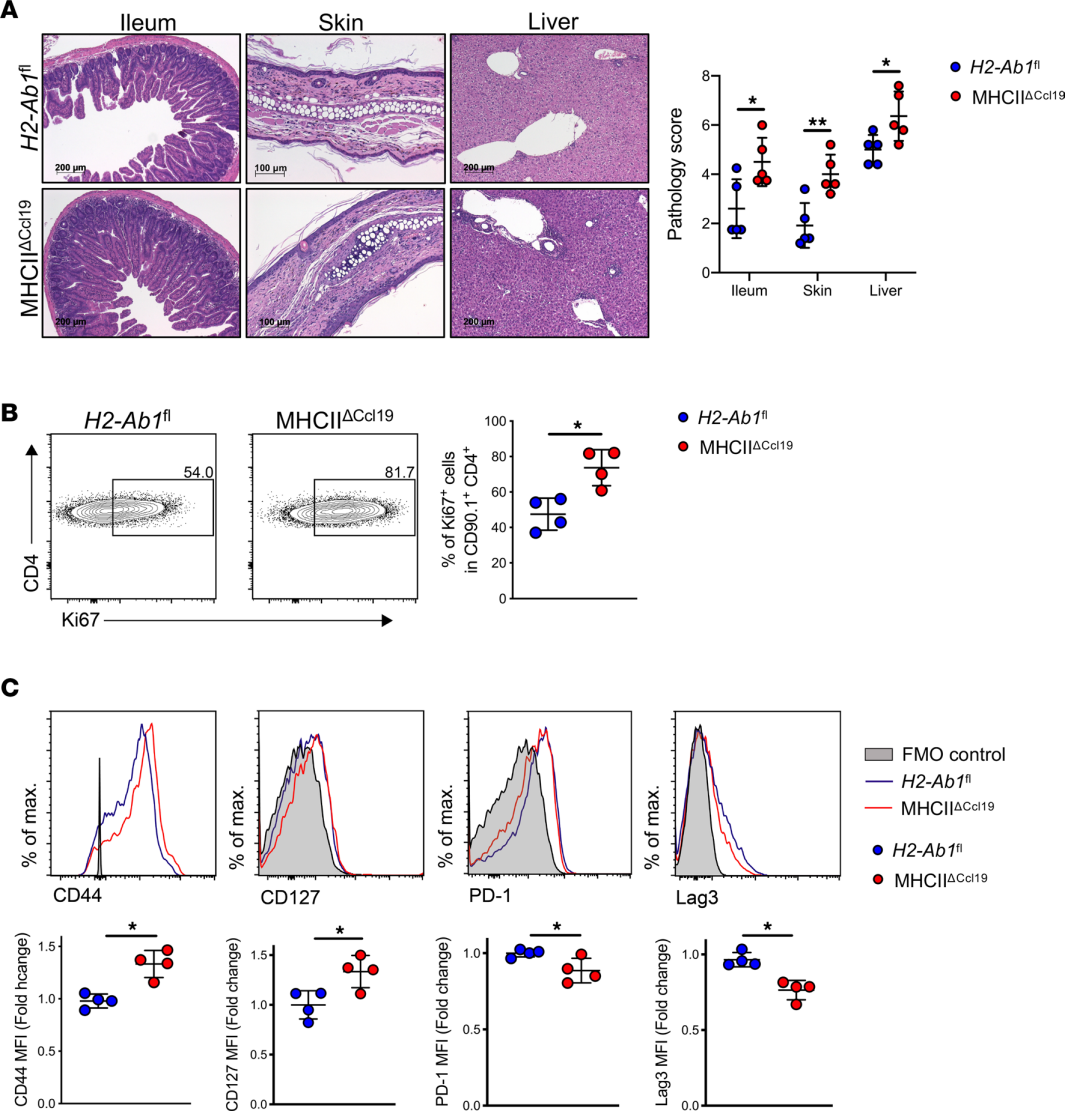
MHCII^ΔCcl19^ mice develop exacerbated aGvHD. (**A**) Photomicrographs depicting typical morphology in ileum, skin, and liver and summarizing histological pathology score at day 60 of allo-HCT. (**B**) Frequency of Ki67^+^ donor allogeneic CD4^+^ T cells (CD90.1^+^CD4^+^). (**C**) Expression of CD44, CD127, PD-1, and Lag3 in normalized MFI on donor allogeneic CD4^+^ T cells (CD90.1^+^CD4^+^) at day 60 of allo-HCT in spleen. Data were pooled from 2 experiments, with 1 data point representing 1 mouse. Unpaired nonparametric Mann-Whitney *U* test was used; data are shown as mean± SD, *P < 0.05 and **P < 0.01.

When we assessed the phenotype at later stages of aGvHD (day 60 of allo-HCT), MHCII^ΔCcl19^ recipients developed exacerbated aGvHD (Figure 3I) with significantly higher pathological scores in GvHD target organs (ileum, liver, and skin) (Figure 5A), higher frequency of proliferating allogeneic CD4^+^ T cells (Figure 5B), higher expression of effector molecules (CD44 and CD127), and downregulated T cell exhaustion molecules (PD-1 and Lag3) in spleen (Figure 5C). Taken together, these experiments revealed that MHCII on FRCs dampens donor allogeneic CD4^+^ T cell alloreactivity and regulates donor Tregs in the effector phase of GvHD.

**Figure 6:**
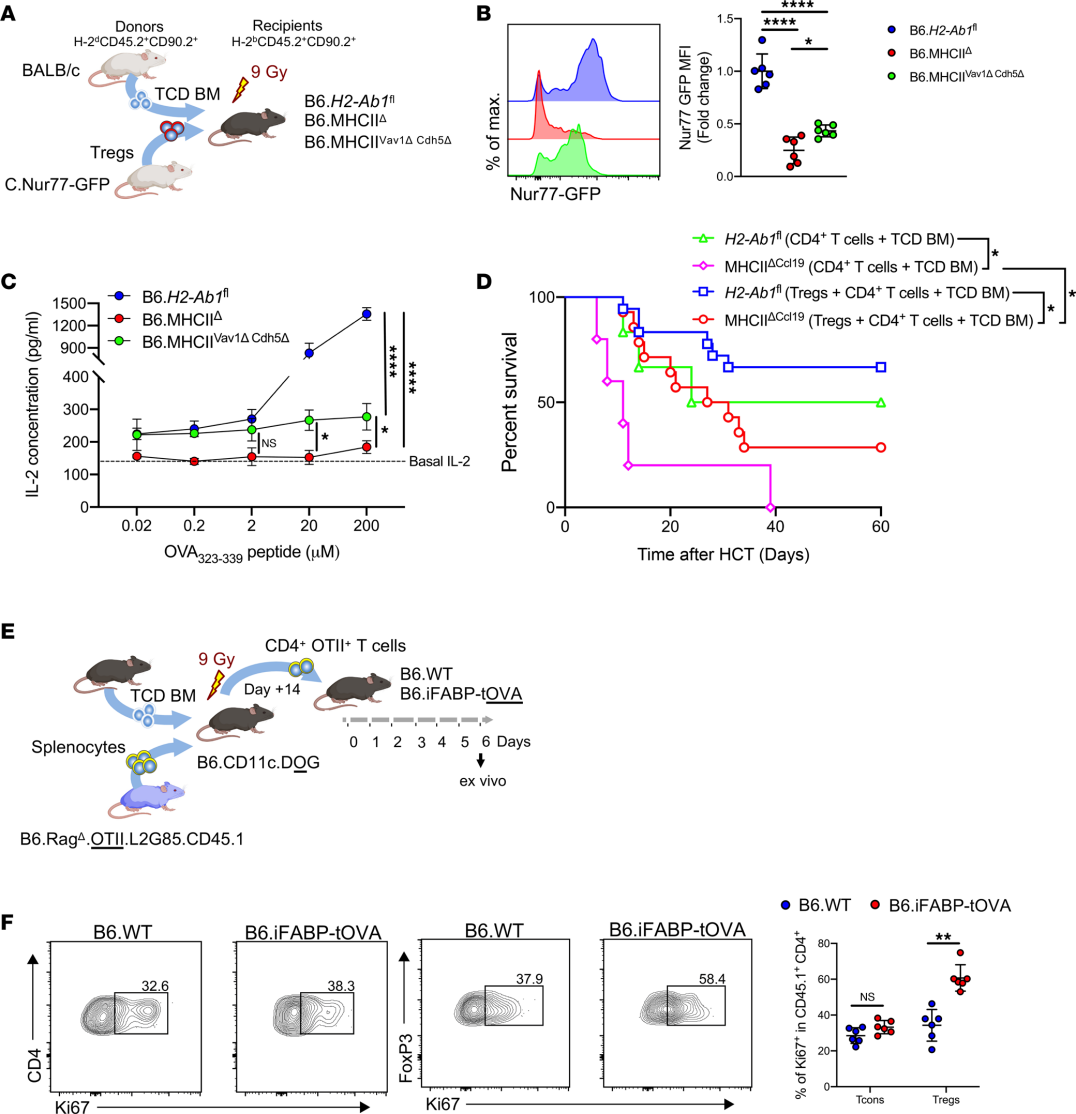
MHCII on FRCs maintains functional CD4^+^FoxP3^+^ Tregs in the effector phase of aGvHD. (**A**) MHCII-competent B6.*H2-Ab1*^fl^, MHCII-deficient B6.MHCII^Δ^, and B6.MHCII^Vav1Δ^ ^Cdh5Δ^ (lacking MHCII expression on all hematopoietic and endothelial cells) recipients were myeloablatively irradiated with 9 Gy and i.v. transplanted with 1 × 10^6^ BALB/c.Nur77-GFP Tregs and 5 × 10^6^ BALB/c WT TCD-BM. (**B**) Expression of Nur77-GFP as normalized MFI on donor CD3ε^+^CD4^+^H-2K^d^ Tregs on day 3 of allo-HCT in LNs. (**C**) LNs from B6.*H2-Ab1*^fl^, B6.MHCII^Δ^, and B6.MHCII^Vav1Δ^ ^Cdh5Δ^ were enzymatically digested and magnetically depleted of CD45^+^ cells, and 2 × 10^4^ cells were cocultured with 8 × 10^4^ BO-97.10 T hybridoma cells expressing an OVA-specific TCR along with titrated amounts of the OVA peptide 323–339. After 24 hours, supernatant was collected and analyzed for IL-2 production by ELISA. Data pooled from 2 experiments, with 1 data point representing 1 mouse. Two-way ANOVA with Tukey test was used; data are shown as mean± SD. *P < 0.05 and ****P < 0.0001. (**D**) Survival of myeloablatively irradiated (9 Gy) B6.*H2-Ab1*^fl^ and B6.MHCII^ΔCcl19^ mice, transplanted with 5 × 10^6^ TCD BM and 6 × 10^5^ CD4^+^ T cells with/without 3 × 10^5^ Tregs mice. Data are illustrated on Kaplan-Meier curve. (**E**) Experimental strategy: B6.CD11c.DOG recipients were myeloablatively irradiated with 9 Gy and i.v. transplanted with 5 × 10^6^ splenocytes and 5 × 10^6^ BM cells from B6.Rag^Δ^.OTII.L2G85.CD45.1 and B6.WT mouse, respectively. At day +14 of syn-HCT, mice were euthanized and CD4^+^ T cells were enriched from the spleen and LNs. Subsequently 1 × 10^7^ enriched CD4^+^ T cells were transplanted into B6.WT and B6.iFABP-tOVA mice. (**F**) Frequency of Ki67^+^ Tcons and Tregs at day +6 of adoptive transfer of OT-II CD4^+^ T cells in B6.WT and B6.iFABP-tOVA mice in spleen. Data pooled from 2 experiments, with 1 data point representing 1 mouse. Unpaired nonparametric Mann-Whitney *U* test was used; data are shown as mean± SD. **P < 0.01.

As *Ccl19*-expressing FRCs could modulate donor Tregs, we further asked whether MHCII on FRCs can induce T cell receptor (TCR) signaling on Tregs. Therefore, we utilized *Nur77*-eGFP reporter mice, which express eGFP upon TCR stimulation (51, 52), as allo-HCT donor mice. Transfer of enriched Tregs (Supplemental Figure 7A) from BALB/c.Nur77-eGFP donors into myeloablatively conditioned MHCII^ΔVav1^ ^ΔCdh5^ hosts lacking MHCII expression on all hematopoietic and endothelial cells — thus, exclusively expressing MHCII on FRCs in SLOs (Figure 6A) — induced *Nur77*-eGFP expression that was significantly higher to complete MHCII-KO mice (MHCII^Δ^) (Figure 6B and Supplemental Figure 7B). To corroborate these findings, we cocultured the CD45^–^ fraction of LN cells from *H2-Ab1*^fl^, MHCII^Δ^, and MHCII^ΔVav1^ ^ΔCdh5^ mice with BO-97.10 T hybridoma cells (53) in the presence of OVA peptide 323–339 and measured the secretion of IL-2 as a read-out for MHCII/TCR engagement. As expected, *H2-Ab1*^fl^ cocultures produced the highest IL-2 amounts due to the presence of contaminating CD45^+^ in the culture. Nevertheless, cultures of MHCII^ΔVav1^ ^ΔCdh5^ LN cells, in which only FRCs could present the OVA peptide 323–339 via MHCII, had significantly higher levels of IL-2 compared with MHCII^Δ^ cultures (Figure 6C). Moreover, on similar lines, culturing of the CD45^–^ fraction of LN cells from B6.iFABP-tOVA mice with BO-97.10 T hybridoma cells resulted in significantly higher IL-2 production than in WT control (Supplemental Figure 7, C and D). Taken together, these findings confirm that MHCII expression on FRCs results in downstream TCR signaling on CD4^+^ T cells.

To assess the relevance of MHCII presentation for in vivo Treg function after allo-HCT, we cotransferred Tregs at the time of allo-HCT with BM and CD4^+^ Tcon at a ratio of 1:2 in both control and MHCII^ΔCcl19^ recipients. Despite the concomitant adoptive Treg transfer at the time of allo-HCT, MHCII^ΔCcl19^ recipients succumbed to aGvHD with significantly reduced survival compared with that of *H2-Ab1*^fl^ WT littermates that received Tregs. Moreover, transfer of Tregs in MHCII^ΔCcl19^ mice only partially protected them from aGvHD, and the survival rate was even lower when compared with *H2-Ab1*^fl^ WT littermates that had not received additional Tregs (Figure 6D). To further dissect the role of MHCII expression by FRC in the maintenance of donor Tregs that resulted in the mitigation of aGvHD, we again utilized an antigen-specific approach employing OT-II TCR transgenic mice (54) on a *Rag1*-deficient background. Syngeneic HCT (syn-HCT) of BM and splenocytes expressing firefly luciferase^+^CD4^+^ OT-II T cells from B6 and B6.Rag^Δ^.OTII.L2G85.CD45.1 mice (Figure 6E and Supplemental Figure 8A), respectively, transferred into myeloablatively irradiated B6.CD11c.DOG mice resulted in activation and expansion of OT-II CD4^+^ T cells, which became evident by bioluminescence imaging (BLI) at day 14 after transplantation (Supplemental Figure 8B). Following their expansion until day 14 after transfer, activated OT-II CD4^+^ T cells were magnetically enriched from the SLOs of B6.CD11c.DOG mice; their transfer into B6.iFABP-tOVA mice resulted in a significant increase in Treg proliferation (CD45.1^+^CD4^+^FoxP3^+^) but not that of Tcons (CD45.1^+^CD4^+^) when compared with control mice lacking antigen expression in the spleen (Figure 6F and Supplemental Figure 8C) and, to a lesser extent, in pLNs and mLNs (Supplemental Figure 8, D and E). However, proliferation of OT-II Tregs in IEL fraction and LP remained unchanged (Supplemental Figure 8F).

Together, these data reveal that MHCII expression on FRCs promotes the expansion of antigen-specific Tregs and controls T cell alloreactivity in the effector phase of GvHD.

## Discussion

Tregs are pivotal in the regulation of aGvHD, whether used as a preemptive therapy (55–59) or for the treatment of established aGvHD (60–62). In recent years, preclinical and clinical studies have demonstrated the therapeutic potential of Tregs by targeting receptors that regulate their proliferation and function (63–69). However, the cellular players essential for Treg maintenance after allo-HCT have not yet been explored. Our results provide evidence for an in vivo role of MHCII expression on Ccl19^+^ FRCs to maintain Tregs and regulate alloimmune responses in aGvHD.

FRC subsets play a critical role in promoting T cell responses by providing DLL1/4 Notch ligands (33), and secretion of IL-6 enhancing their survival, metabolism, and capacity to differentiate into tissue-resident memory populations (24). In a different context, FRCs have also been shown to suppress T cell proliferation by IFN-γ–dependent upregulation of nitric oxide synthase 2 (NOS2) in a cell-contact–dependent manner (19, 20, 70).

Ccl19*^+^* FRCs have been cell traced to be located within LNs, spleen, and PPs, where naive donor T cells directly home to after allo-HCT (71). Previously, it has been demonstrated that fibroblastic SCs express MHCII via the pIV of the CIITA, the master regulator of MHCII expression, and can acquire peptide-MHCII complexes from DCs inducing CD4^+^ T cell dysfunction (26, 27). Here, we found that myeloablative irradiation activated the FRCs, resulting in upregulation of costimulatory receptors in vitro and in vivo. Surprisingly, postirradiation MHCII expression on FRCs was downregulated within 24 hours, which coincided with loss of DCs in spleen and mLNs, suggesting that irradiation indirectly influences MHCII presentation on FRCs by depleting radiosensitive DCs. We further demonstrated that FRCs at steady state, as well as 48 hours after allo-HCT, could process and degrade DQ-OVA, and even in the absence of MHCII transfer from DCs to FRCs in (MHCII^ΔVav1^ mice), DQ-OVA processing and degradation was not abrogated. These results indicate that FRCs could upregulate costimulatory molecules and process exogenous antigens through MHCII machinery. scRNA-Seq revealed that SCs of the LNs have a distinct expression profile of molecules involved in MHCII-mediated antigen processing and presentation, with Ccl19^+^ SC subsets showing comparatively higher expression than all other subsets. Both *ctsl* and *ctss* play crucial roles in the degradation of li, and their expression has been linked to positive (tolerance) (41) and negative (immunity) (42) selection of CD4^+^ T cells. Here, we detected stable yet differential expression of *Ctsl* on all subsets of SCs, whereas *Ctss* was exclusively expressed on Ccl19^+^ SCs in the LNs. Moreover, molecules that are crucial in tagging antigen for macroautophagy were highly expressed on Ccl19^–^ SC subsets (PvCs, CD34^+[Ackr3+]^, CD34^+[Aldh1a2+]^, Inmt^+^). Taken together, these data suggest that FRCs can directly modulate CD4^+^ T cell response in aGvHD.

However, surprisingly, MHCII loss of function on Ccl19^+^ FRCs only resulted in moderate but not significant alteration of alloreactive CD4^+^ T cell activation in the initiation phase of aGvHD. This reveals that alloreactive donor T cells require critical Notch signals (33) but not alloantigen presentation from FRCs to drive aGvHD alloresponses. Utilizing a TCR transgenic model expressing tOVA expressed under iFABP promotor (48), Lee and colleagues identified an important role of FRCs in MHCI-dependent OVA-specific CD8^+^ T cell (OT-I) activation and proliferation in LNs and PPs (72), but they also demonstrated a subsequent loss of these antigen-specific CD8^+^ T cells after initial proliferation, which suggests also a tolerogenic capacity of FRCs. In MHCII-driven CD4^+^ T cell responses, we found that antigen-specific OT-II CD4^+^ T cells failed to be activated and proliferate in myeloablatively irradiated iFABP-tOVA mice. Taken together, although FRCs can upregulate costimulatory molecules and process exogenous antigens, they could not prime and activate polyclonal and antigen-specific CD4^+^ Tcon under aGvHD-like inflammatory conditions.

Self-antigen presentation by FRCs via MHCI and MHCII to T cells in the effector phase of aGvHD modulates alloimmunity and delays symptoms of GvHD. GvHD can lead to selective elimination of FRCs, which results in the loss of peripheral tissue–restricted antigen presentation, resulting in the activation of auto-aggressive T cells (30, 31). Consequently, we interrogated the relevance of FRC-restricted MHCII presentation in the effector phase of aGvHD. MHCII^ΔCcl19^ recipients developed severe and accelerated GvHD with higher expression of T cell activation and proliferation markers in SLOs. Allogeneic CD4^+^ T cells in MHCII^ΔCcl19^ mice downregulated genes involved in aerobic glycolysis, suggesting that, indeed, CD4^+^ T cells in MHCII^ΔCcl19^ have transitioned into the memory phase in which alloreactive T cell biosynthesis is primarily maintained by fatty acid oxidation. Conversely, CD4^+^ T cells in *H2-Ab1*^fl^ littermate control animals were still expanding during the effector phase of GvHD, primarily consuming glutamine and using aerobic glycolysis as their primary metabolic source (73). Since RNA-Seq and flow cytometry demonstrated a loss of CD4^+^ iNKT cells in MHCII^ΔCcl19^ animals, which can regulate GvHD through expansion of donor Tregs (74, 75), it is tempting to speculate that their dysregulation influences GvHD outcome in MHCII^ΔCcl19^ mice. Indeed, we observed reduced numbers of FoxP3^+^ Tregs that downregulated expression of Helios in SLOs of MHCII^ΔCcl19^ mice. Our findings are consistent with observations from autoimmune disease models where FRCs indirectly restrain aberrant CD4^+^ T cell activation via MHCII presentation of self-antigen to induce proliferation of FoxP3^+^ Tregs (27, 29) and de novo conversion of Tregs (39, 76, 77) in the mLNs under homeostatic conditions. Consistent with the idea that FRCs form important hubs to maintain functional Tregs after allo-HCT, on day 60 after allo-HCT, as a late stage of the aGvHD effector phase, an exacerbated aGvHD phenotype in MHCII^ΔCcl19^ mice became even more prominent. Increased expression of effector molecules and downregulation of T cell exhaustion markers on alloreactive CD4^+^ T cells led to overall poor survival. Directly addressing the role of FRC-restricted MHCII presentation to donor Tregs, we uncovered that adoptively transferred Tregs in MHCII^ΔCcl19^ recipient mice with MHCII-deficient FRCs clearly failed to protect against GvHD in contrast to WT *H2-Ab1*^fl^ littermate control recipients. Along these lines, we showed that MHCII on FRCs induced downstream TCR signaling in Tregs and that MHCII antigen presentation by FRCs selectively promoted the proliferation of activated OVA-specific Tregs but not OVA-specific CD4^+^ Tcon. These data support a direct role of FRCs within SLOs in maintaining antigen-specific Tregs.

Treg therapy has been proven as an efficient strategy to mitigate GvHD, while allowing for the desired GvL effect in preclinical mouse models, as well as in clinical trials (56, 58, 60, 61, 78–80). The data presented here identify FRCs as important immune-regulatory hubs that maintain Tregs through MHCII-mediated mechanisms during the effector phase of acute GvHD. Whether adoptive transfer of FRCs or direct targeting of FRCs with small molecules or biologicals can foster Treg maintenance and function deservers further exploration. In conclusion, FRCs should be considered attractive therapeutic targets to regulate T cell alloreactivity in aGvHD after allo-HCT.

## Methods

### Mice.

C57BL/6 (B6, H-2^b^), FVB/N (H-2^q^), and BALB/c (C, H-2^d^) mice were purchased from Charles River Laboratories and Janvier Laboratories. B6.iFABP-tOVA mice were a gift from Vaiva Vezys (University of Minnesota, Minneapolis, Minnesota, USA) and have been previously described (48). C57BL/6-background R26-stop-EYFP, *H2-Ab1*^fl^, *Vav1*-iCre, *Cdh5*-Cre and MHCII^Δ^ (null) mice were purchased from The Jackson Laboratory. B6.CD11c.DOG (81) mice were provided by Günter J. Hämmerling (German Cancer Research Center, Heidelberg, Germany). BAC-transgenic B6-background *Ccl19*-Cre mice have been previously described (46). *Ccl19*-Cre mice were crossed with R26-stop-EYFP to generate FRCs reporter; subsequently, this mouse was crossed with *H2-Ab1*^fl^ mice to generate MHCII^ΔCcl19^ mice. Moreover, with *H2-Ab1*^fl^ mice was crossed with *Vav1*-iCre and *Cdh5*-Cre mice to generate MHCII^ΔVav1^ and MHCII^ΔCdh5^ mice, respectively. Subsequently, MHCII^ΔVav1^ and MHCII^ΔCdh5^ were crossed to generate animals deficient in MHCII in cells of hematopoietic and endothelial lineages. BALB/c.Nur77-eGFP (C, H-2^d^) mice were a find gift from Kristin A. Hogquist (University of Minnesota) and have been previously described (51). FVB/L2G85 (H-2^q^, CD90.1, CD45.1) expressing firefly luciferase were generated as described previously (35, 82). B6.Rag^Δ^.OTII.L2G85.CD45.1 were generated by crossing Rag^Δ^ mice (83) with mice expressing OVA-specific TCR on CD4^+^ T cells (54) crossed to B6 mice harboring the CAG-luc-eGFP L2G85 transgene carrying *Ptprc*^a^ (82, 84). B6.Rag^Δ^.OTI.L2G85.CD45.1 were generated by crossing Rag^Δ^ mice (83) with mice expressing OVA-specific TCR on CD8^+^ T cells and (85) crossed to B6 mice harboring the CAG-luc-eGFP L2G85 transgene carrying *Ptprc*^a^ (82, 84).

Mice were kept in pathogen-free conditions in individually ventilated cages (IVCs) at the Center for Experimental Molecular Medicine (ZEMM), Würzburg, Germany.

### Cell culture.

Mouse iFRCs was a gift from Sophie Acton (University College London, London, United Kingdom) (15). iFRCs were cultured in DMEM supplemented with 1% penicillin-streptomycin, 1% insulin, transferrin, selenium solution (ITS), 10% FCS (all from Thermo Fisher Scientific). BO-97.10 T hybridoma cells (53) were cultured in RPMI supplemented with 1% penicillin-streptomycin and 10% FCS.

### Antigen processing assay.

Purity of cells sorted by FACS was >95%. Sorted cells were incubated with 10 μg/mL BODIPY-conjugated DQ-OVA (Invitrogen) in RPMI-1640 media supplemented with 1% penicillin-streptomycin and 10% FCS (Thermo Fisher Scientific) at 4°C and 37°C for 3 hours. Incubation was followed by processed DQ-OVA analysis on Attune NxT flow cytometer (Thermo Fisher Scientific) under the blue laser (488 nm), BL-1.

### HCT.

Sex-matched, 8- to 12-week-old (B6, H-2^b^) recipient mice received myeloablative total body irradiation (TBI) of 9 Gy using a Faxitron CP-160 x ray irradiation system (Faxitron X-Ray). Within 4 hours after TBI, mice were i.v. injected (retro-orbitally) with 5 × 10^6^ FVB/N T cell–depleted (TCD) BM cells for hematopoietic reconstitution. T cells from the BM were depleted using CD90.1 MicroBeads (Miltenyi Biotec) following manufacturer instructions. To induce aGvHD, allogeneic enriched 6 × 10^5^ CD4^+^ T cells from FVB/N or FVB.L2G85 mice were coinjected i.v. T cells were purified from the spleen using Dynabeads Untouched Mouse CD4^+^ Cells Kit (Invitrogen), whereas Tregs were purified from the spleen using Dynabeads Regulatory CD4^+^/CD25^+^ T Cell Kit (Invitrogen), according to manufacturer instructions. Cell purity was accessed with flow cytometry (>95% purity).

### Flow cytometry.

In vitro–cultured and single-cell suspensions of primary mouse cells were incubated with normal rat serum (NRS) (1 part NRS to 20 parts PBS) for 5 minutes at 4°C to block unspecific binding to Fc receptors. Cells were stained with fluorochrome-labeled antibodies for 30 minutes at 4°C. To exclude dead cells from the analysis, cells were costained with LIVE/DEAD Fixable Violet Dead Cell Stain Kit (Invitrogen). Used antibodies are listed in Table 1. Data were acquired with a BD FACS Canto II flow cytometer (BD Biosciences) or Attune NxT flow cytometer (Thermo Fisher Scientific). Acquired cytometry data were analyzed with FlowJo version 10 software (Tree Star Inc.).

To compensate for the spill over in the emission spectra for each fluorochrome, UltraComp eBeads Compensation Beads (Invitrogen) were individually stained with the single-fluorochrome–labeled antibodies used in the multicolor-antibody panels. This compensation procedure allowed for calculating and subtracting the appropriate overlap to yield the specific signal intensity for each fluorochrome. To set the gates in multicolor-stained samples, the fluorescence minus 1 (FMO) method (86) was performed.

For FACS, cells isolated from mouse tissue were antibody stained and sorted with a FACS Aria III (BD Biosciences). CD4^+^ T cells, DCs, and FRCs were all sorted with a 100 μm nozzle into ice-cold cell culture medium or lysis buffer depending on downstream application.

### Reverse transcription PCR (RT-PCR).

Expression of OVA on FRCs from B6.iFABP-tOVA mice was assessed after FACS of mLN FRCs from B6.WT and B6.iFABP-tOVA into lysis buffer (purity > 95%). RNA was isolated using RNeasy Mini Kit (Qiagen), quantified and cDNA synthesized with the RevertAid first strand cDNA synthesis kit (Thermo Fisher Scientific), and PCR was performed by the KAPA HotStart Mouse Genotyping Kit (Sigma-Aldrich) using the primer pairs: Gapdh, 5′-AGTATGACTCCACTCACGGC-3′ plus 5′-ATGTTAGTGGGGTCTCGCTC-3′; Ova, 5′-GCTGCAGATCAAGCCAGAGAGC-3′ plus 5′-ATTGATTTCTGCATGTGCTGC-3′.

### Quantitative PCR (qPCR).

Expression of CIITA pI and pIV was performed on magnetic enriched cell sorting (MACS) enriched DCs using CD11c MicroBeads UltraPure, mouse (Miltenyi Biotec) following manufacturer’s instructions, whereas FRCs were FACS sorted into lysis buffer (purity > 95%). RNA was isolated using RNeasy Mini Kit (Qiagen), quantified and cDNA synthesized with the RevertAid first strand cDNA synthesis kit (Thermo Fisher Scientific) followed by qPCR was performed by SsoAdvanced Universal SYBR Green Supermix (Bio-Rad) using the primer pairs: CIITA pI, 5′-CAGGGACCATGGAGACCATAGT-3′ plus 5′-CAGGTAGCTGCCCTCTGGAG-3′; CIITA pIV, 5′-CAGCACTCAGAAGCACGGG-3′ plus 5′-ATCCATGGTGGCACACAGACT-3′.

### RNA-Seq and analysis.

*H2-Ab1*^fl^ and MHCII^ΔCcl19^ mice were transplanted with 5 × 10^6^ TCD BM and 6 × 10^5^ CD4^+^ T cells from FVB/N. On day 30, spleens were processed and magnetically enriched for CD4^+^ T cells using Dynabeads Untouched Mouse CD4^+^ Cells Kit (Invitrogen) according to manufacturer instructions. Enriched cells were stained with LIVE/DEAD Fixable Violet Dead Cell Stain Kit (Invitrogen) to exclude dead cells and CD90.1^+^CD4^+^ staining to identify donor alloreactive CD4^+^ T cells. Total viable, CD90.1^+^CD4^+^ cells were sorted using a 100 μm nozzle in 150 μL of lysis buffer and stored on dry ice for further processing.

RNA was isolated using the PicoPure RNA isolation kit (Thermo Fisher Scientific) following manufacturer protocol. Isolated RNA was quantified on 2100 Bioanalyzer instrument (Aligent). cDNA synthesis and subsequent library preparation were performed with 1 ng of RNA using a NEBNext Single Cell/Low Input RNA Library Prep kit for Illumina following manufacturer protocol. Libraries were sequenced on Illumina NextSeq 500 system as single-end sequencing and 75 bp read length.

Read quality of sequenced libraries was evaluated with FastQC. Sequencing reads were aligned to the reference mouse genome assembly GRCm38 using STAR (version 2.7.0e) (87). Reads aligned to annotated genes were quantified with *htseq-count* (88). Protein-coding genes with at least 5 reads in at least 2 replicates were included in the analysis. The calculated read counts were further processed with DESeq2 (version 1.26.0) for quantification of differential gene expression (89). Raw read counts were converted to reads per kilobase per million mapped reads (RPKM) values. Genes were considered as differentially expressed at log_2_ (fold change) > 0.75 and an adjusted *P* < 0.05. Heatmaps were visualized using pheatmap (version 1.0.12)*,* and fold change and volcano plots were visualized using ggplot2 (version 3.3.2). RNA-Seq data has been uploaded to the Gene Expression Omnibus (GEO) database under accession no. GSE168114.

### Single-cell RNA-Seq data analysis.

Raw count matrices were accessed from a publicly available data set (https://www.ncbi.nlm.nih.gov/geo/query/acc.cgi?acc=GSE116633) with GEO accession no. GSE116633 which was published by Pezoldt and colleagues (39). Quality control analysis, filtering, and normalization of scRNA-Seq data were performed using SCANPY toolkit (90) in python. Cells with less than 500 and more than 5,000 detected genes per cell were filtered. To remove low-quality or dead cells, the fraction of mitochondrial genes transcription was calculated, and cells with more than 7.5% of mitochondrial genes were eliminated from the downstream analysis (91). Furthermore, genes that appeared in less than 3 cells were filtered out. After normalizing the counts of each cell to the natural logarithm, we selected 3,000 variable genes and regressed out the effect of total counts per cell and percentage of mitochondrial genes expressed. Finally, each gene counts (https://www.cureffi.org/2013/09/12/counts-vs-fpkms-in-rna-seq/) were scaled to a unit variance.

To explore the main axis of data variation, selected variable genes were used for principal component analysis (PCA) and dimensional reduction of data. By choosing 40 as number of PCs and 20 as number of neighbors, we embed the data in 2 dimensional UMAP manifold. To classify cells based on similarity in gene expression signatures, leiden clustering with resolution = 1 was employed. We removed endothelial cells with expression level of *Pecam* (CD31 cell surface receptor) > 1.5. The remaining 9,278 cells from 2 mLNs and 2 peripheral LNs were reclusterd with leiden clustering (resolution = 1).

To annotate each cluster, we plotted the mean expression level of a set of genes in each cluster as a dot plot. Furthermore, we used a DEG set in each cluster versus other cells for confirmation of cell annotation.

### IL-2 assays.

IL-2 concentration in cell culture media secreted by BO-97.10 hybridomas upon coculture with CD45^–^ fraction of LN was measured with an IL-2 mouse ELISA kit (Thermo Fisher Scientific) following manufacturer’s instructions. For flow cytometric measurement of IL-2, the plates were centrifuged after 16 hours of coculture, and media were replaced with complete RPMI (cRPMI) supplemented with phorbol-12-myristate 13-acetate (PMA), ionomycin, and Brefeldin A for 8 hours, followed by antibody staining.

### BLI.

In vivo BLI was performed using the IVIS Spectrum CCD-imaging system (PerkinElmer) as previously described (92). Briefly, mice were anesthetized with an i.p.-injected mixture of ketamine (50 μg/g body weight) and xylazine (5 μg/g body weight) in PBS in a total volume of 10 μL/g body weight. D-Luciferin was injected in a concentration of 300 mg/kg of body weight, and images were taken 10 minutes after the injection and allowed the identification of T cell proliferation and migration. Alternatively, mice were i.p. injected with 300 mg/kg of D-Luciferin and anesthetized with 2% isoflurane in O_2_. After 10 minutes, the bioluminescence signal was acquired with an IVIS Spectrum (PerkinElmer). To perform ex vivo imaging, mice were injected with the same mixture of anesthetic and D-Luciferin, and 10 minutes after injection, mice were euthanized, and organs were removed within 4 minutes. Ex vivo images provided higher resolution of selective organ signal distribution. Imaging data were analyzed on Living image 4.5.5 (PerkinElmer) software.

### Histopathological analysis.

Ileum, liver, and skin were fixed in 4% PFA, paraffin embedded, sectioned, and stained with H&E. Slides were examined by a pathologist who was blinded to experimental history.

### Immunofluorescence staining.

Mice were intravascularly perfused with PBS for 2 minutes and, subsequently, with 4% PFA for 8 minutes. Isolated LNs were further fixed in 4% PFA for 3 hours at room temperature. The LNs were equilibrated at 4°C in 10% sucrose solution overnight, followed by 20% sucrose solution for 4 hours and 30% for 2 hours. LNs were cryoembedded, cut into 7 μm–thick sections on a cryostat (CM1900; Leica Biosystems), and mounted onto frosted slides. The slides were blocked with 2% FCS in PBS for 30 minutes using an avidin/biotin blocking kit (Vector Laboratories). The slides were then incubated with primary antibodies (Table 1) for 1 hour at room temperature and were further incubated for 30 minutes with appropriate secondary antibodies, counterstained with DAPI, and mounted with mounting medium (Vector Laboratories). Images were obtained with a confocal laser-scanning microscope (LSM780; ZEISS) at room temperature and analyzed with IMARIS software v8.1.1 (Bitplane AG).

### Statistics.

Data are shown as mean ± SD. Two groups were compared by 2-tailed unpaired Student’s *t* tests or unpaired nonparametric Mann-Whitney *U* test, and comparison between more than 2 groups was performed by 2-way ANOVA, adjusted for multiple comparisons with Tukey’s multiple-comparison test using GraphPad Prism 8 software. Murine survival experiments and Kaplan-Meier curves were analyzed by log-rank test (Mantel-Cox test). Level of significance was set at *P* < 0.05.

### Study approval.

All animal experiments were approved by local authorities (Regierung von Unterfranken) and complied with German animal protection laws under permit nos. 55.2-2532-2-692-17 and 55.2.2-2532-2-410-85.

## Author contributions

HS designed, planned, and carried out the experiments and analyzed and interpreted the data. ZM and JP analyzed sequencing data. JGV, DDL, JPM, EAV, MAGK, and CG assisted with the experiments. BL provided tools and contributed to manuscript writing. JP and AES planned and performed the scRNA-Seq experiments. AR assisted with RNA-Seq experiments. MBH performed the pathology scoring. NB, MBL, HE, JH, AZ, and BL provided intellectual support and assisted in editing the manuscript. AB designed the study and interpreted the data. HS and AB wrote the manuscript. All authors read and discussed the manuscript.

## Supplementary Material

Supplemental data

## Figures and Tables

**Table 1:**
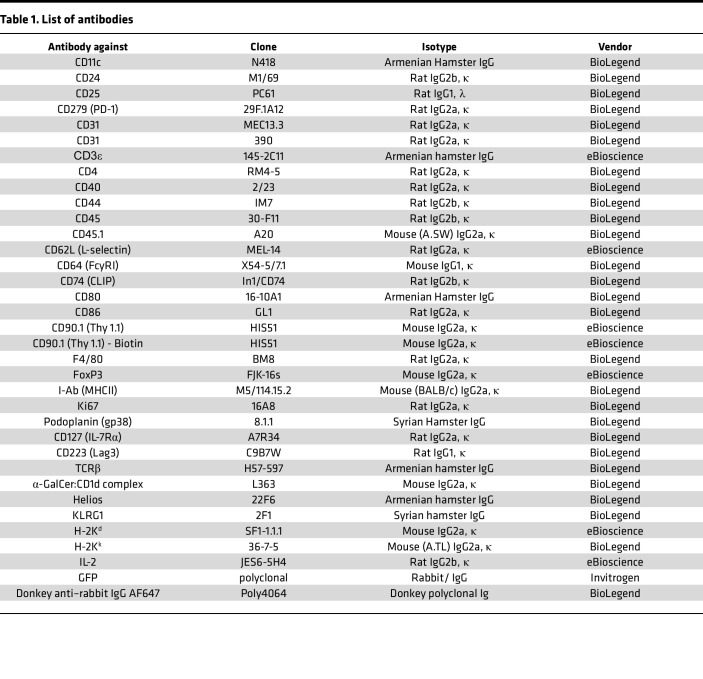
List of antibodies

## Version 1 10/13/2022

In-Press Preview

## Version 2 11/22/2022

Electronic publication
