# Supplementary material for: Fibroblastic reticular cells mitigate acute GvHD via MHCII-dependent maintenance of regulatory T cells
Source: JCI Insight. 2022 Nov 22;7(22):e154250. doi: 10.1172/jci.insight.154250 (PMC9746820; doi:10.1172/jci.insight.154250)
Supplement: Supplemental data [file jciinsight-7-154250-s008.pdf]

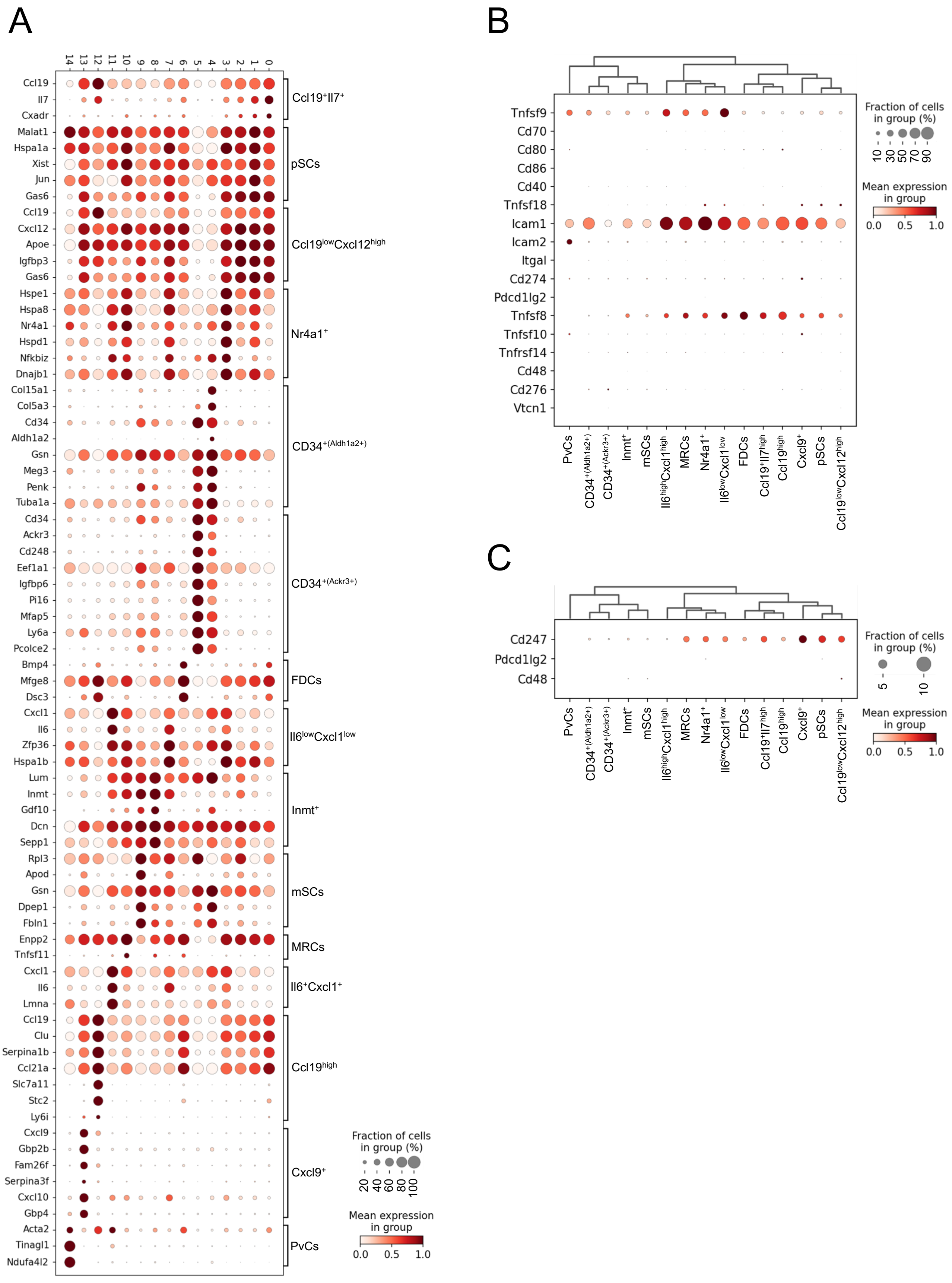

Figure S1: DEGs for cluster annotation of LN derived SCs subset. (**A**) Mean expression level of selected set of genes in each cluster represented as dot plot. We utilized DEGs set in each cluster versus other cells for cluster identification and confirmation of cell annotation. (**B**) Expression of co-stimulatory (**B**) add co-inhibitory molecules on SC subsets.

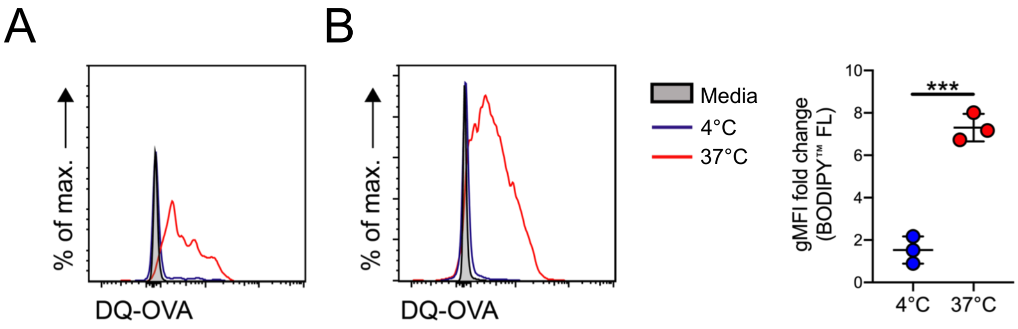

Figure S2: FRCs degrade and process exogenous antigen in the absence of MHCII transfer from DCs. (**A**) FACS sorted lymph node FRCs from B6.MHCII$^{\Delta Vav1}$ mouse incubated with DQ-OVA, (**B**) FACS sorted lymph node FRCs from B6.WT mouse day 2 after allo-HCT, incubated with DQ-OVA for 3 h at 4°C and 37°C, followed by analysis of processed DQ-OVA (blue laser 488 nm, BL-1) on flow cytometer and quantification – normalized to media. Data from one experiment, one data point representing one mouse; Two-tailed unpaired Student's t-tests, (Mean± SD); ****p* < 0.001.

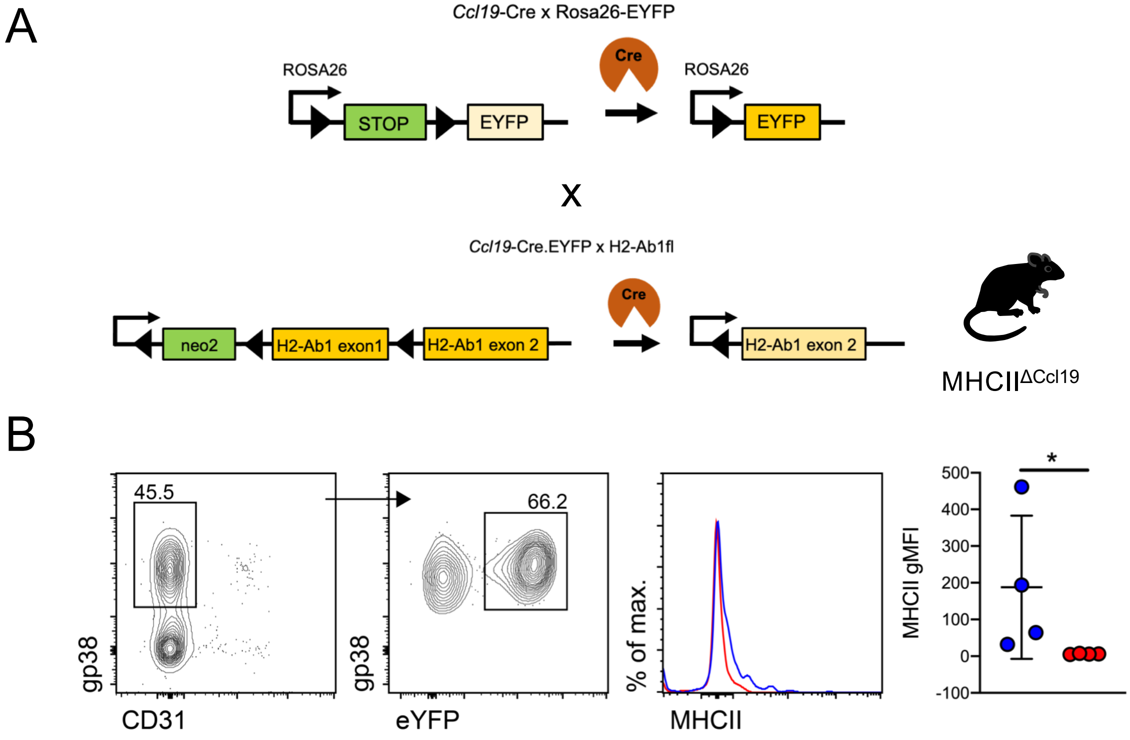

Figure S3: Conditional knock-out of MHC class II on *Ccl19* expressing fibroblastic reticular cells. (**A**) *Cc19*-Cre mice on C57BL/6 background was crossed with Rosa26-EYFP to generate *Cc19*-Cre.EYFP. Subsequently, *H2-Ab1*^{fl} was crossed to *Ccl19*-Cre.EYFP to generate a conditional knock-out of MHCII on FRCs and the mice were designated as MHCII$^{\Delta Ccl19}$. (**B**) Gating strategy: Lymph nodes were enzymatically digested and gated for CD45/Ter119$^-$, gp38$^+$CD31$^-$ population was sub-gated on eYFP expressing *Ccl19*$^+$ cells and assessed for MHCII expression on WT littermates: *H2-Ab1*^{fl} and MHCII$^{\Delta Ccl19}$. Data from one experiment, one data point representing one mouse; Two-tailed unpaired Student's t-tests, (Mean± SD); *p < 0.05.

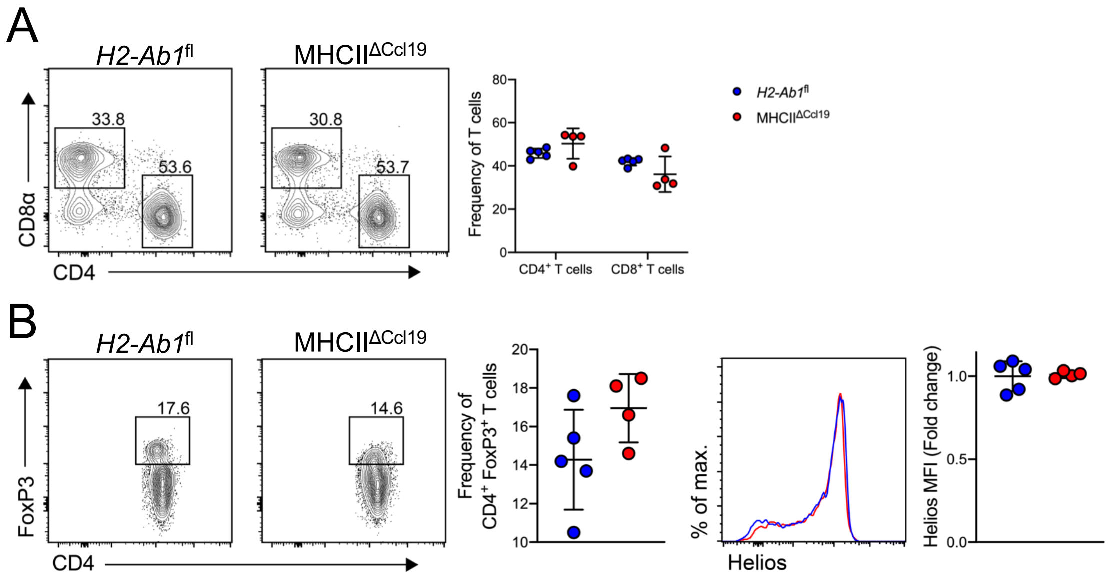

Figure S4: MHCII$^{\Delta Ccl19}$ mice have an intact T cell compartment at steady-state. (**A**) Frequency of CD4^{+} and CD8^{+} T cells in spleen of *H2-Ab1*^{fl} and MHCII$^{\Delta Ccl19}$ mice, pre-gated on singlets, viable and CD3ε^{+}. (**B**) Frequency of Tregs (FoxP3^{+}) and expression of Helios in normalized MFI in spleen of *H2-Ab1*^{fl} and MHCII$^{\Delta Ccl19}$, pre-gated on singlets, viable, CD3ε^{+} and CD4^{+}. Data from one experiment, one data point representing one mouse.

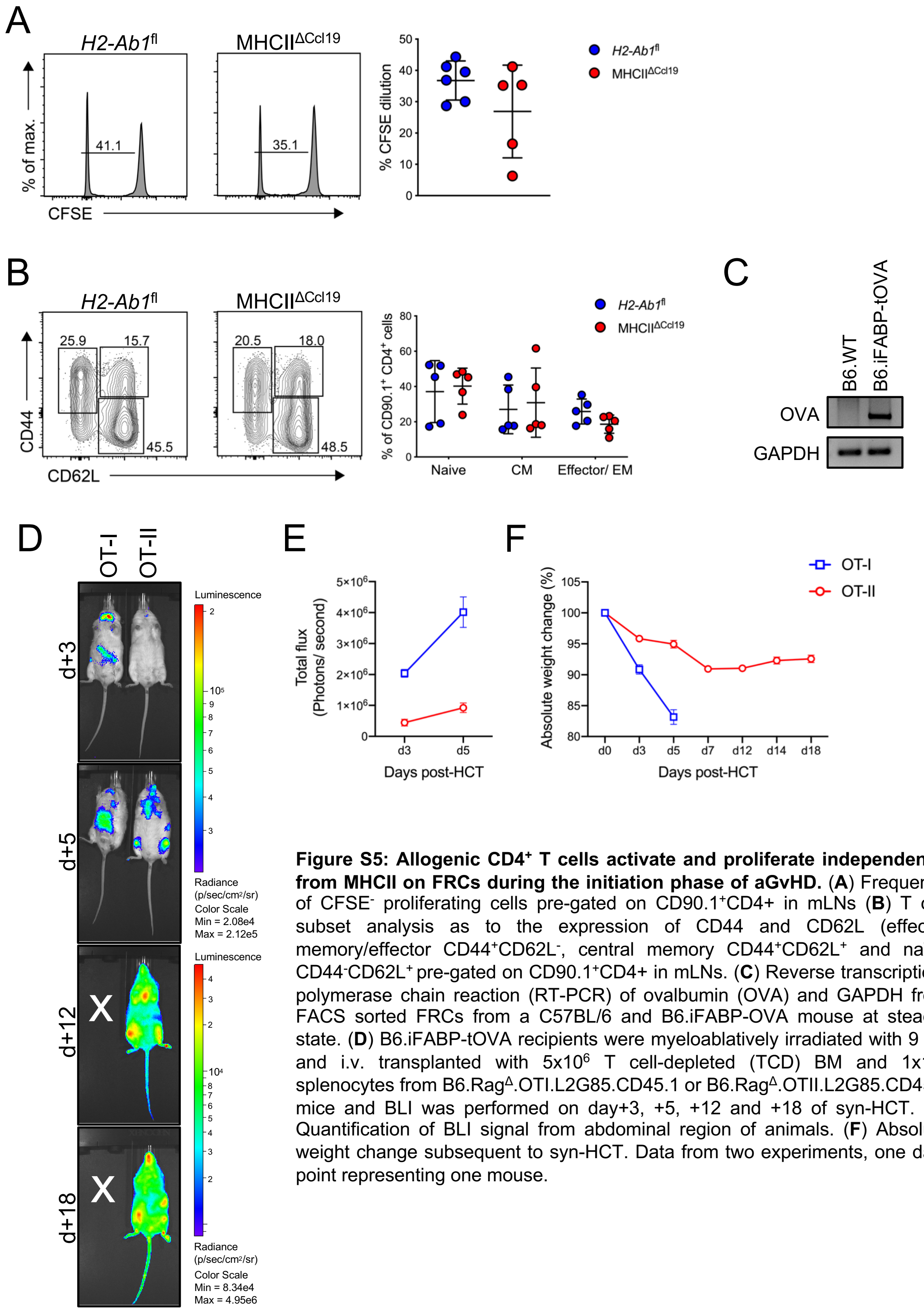

Figure S5: Allogenic CD4$^+$ T cells activate and proliferate independently from MHCII on FRCs during the initiation phase of aGvHD. (**A**) Frequency of CFSE$^-$ proliferating cells pre-gated on CD90.1$^+$CD4+ in mLNs (**B**) T cell subset analysis as to the expression of CD44 and CD62L (effector memory/effector CD44$^+$CD62L$^-$, central memory CD44$^+$CD62L$^+$ and naïve CD44$^-$CD62L$^+$ pre-gated on CD90.1$^+$CD4+ in mLNs. (**C**) Reverse transcription-polymerase chain reaction (RT-PCR) of ovalbumin (OVA) and GAPDH from FACS sorted FRCs from a C57BL/6 and B6.iFABP-OVA mouse at steady-state. (**D**) B6.iFABP-tOVA recipients were myeloablatively irradiated with 9 Gy and i.v. transplanted with 5x10^6 T cell-depleted (TCD) BM and 1x10^7 splenocytes from B6.Rag$^{\Delta}$.OTI.L2G85.CD45.1 or B6.Rag$^{\Delta}$.OTII.L2G85.CD45.1 mice and BLI was performed on day+3, +5, +12 and +18 of syn-HCT. (**E**) Quantification of BLI signal from abdominal region of animals. (**F**) Absolute weight change subsequent to syn-HCT. Data from two experiments, one data point representing one mouse.

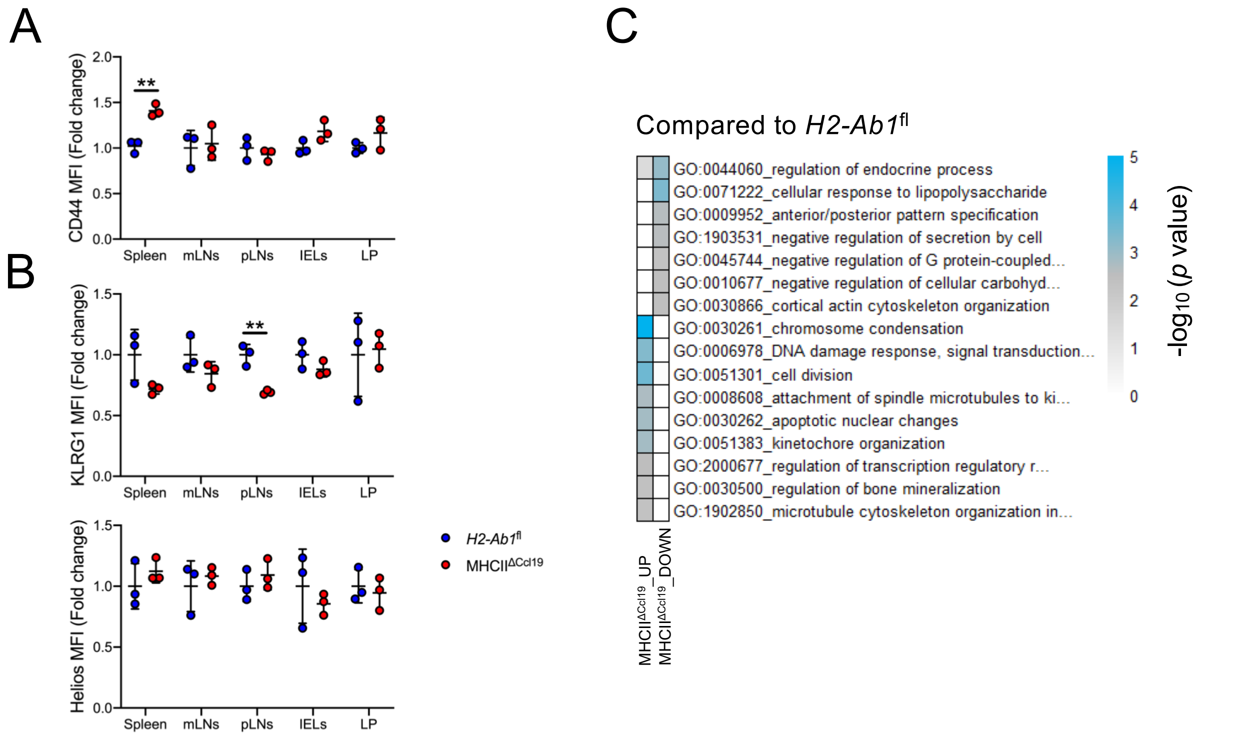

Figure S6: Allogenic CD4$^+$ T cell activation in the effector phase of aGvHD. (**A**) Expression of CD44 on allogenic CD4$^+$ in spleen, mLNs, pLNs, IELs and LP at day +14 of allo-HCT. (**B)** Expression of KLRG1 and Helios on allogenic Tregs in spleen, mLNs, pLNs, IELs and LP at day +14 of allo-HCT. (C) Gene ontology (GO) enrichment analysis on DEGs comparing allogenic CD4$^+$ T cells from MHCII$^{\Delta Ccl19}$ versus *H2-Ab1*^{fl} mice in spleen the at day +30 of allo-HCT. One data point representing one mouse; two-tailed unpaired Student's t-tests, (Mean± SD); ***p* < 0.01.

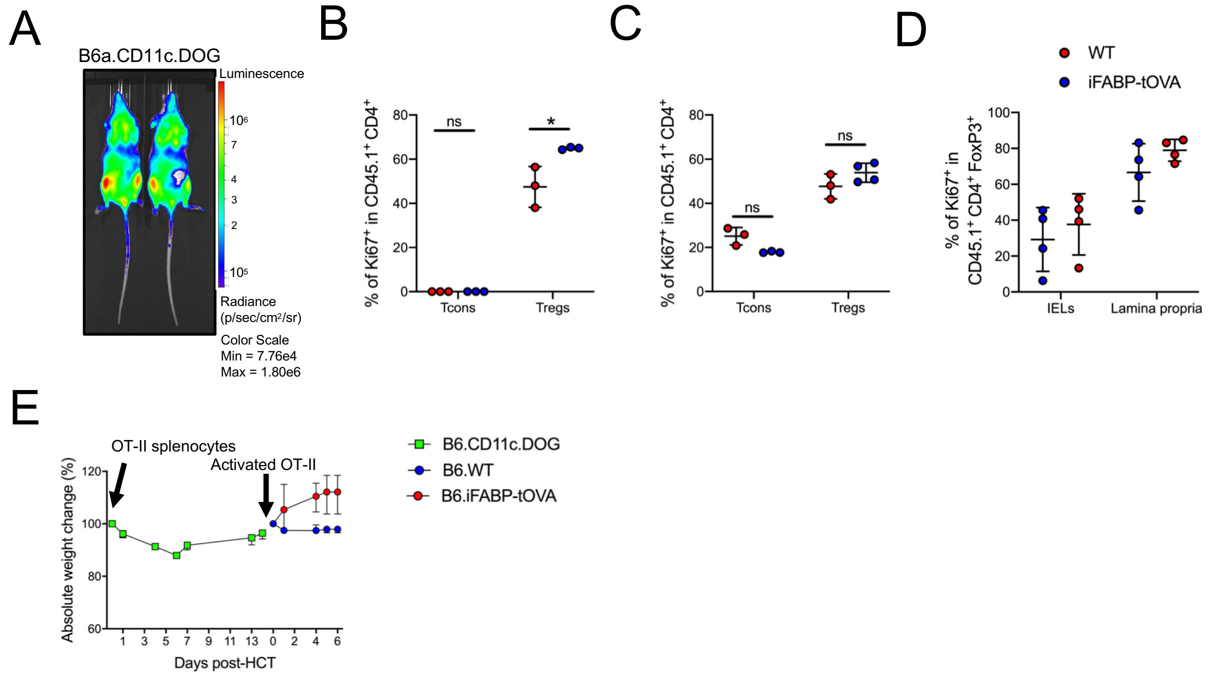

Figure S7: MHCII on FRCs maintains Tregs under inflammatory conditions. (**A**) *In vivo* BLI of OT-II T cells in B6.CD11c.DOG mice at day +14 of syn-HCT. Ki67 expression on donor (CD45.1$^+$CD4$^+$) Tcons and Tregs from B6.WT and B6.iFABP-tOVA in (**B**) pLNs and (**C**) mLNs. (**D**) Ki67 expression on Tregs in IELs fraction and *lamina propria*. (**E**) Absolute weight change of B6.CD11c.DOG, B6.WT and B6.iFABP-tOVA mice during the course of experiment. Two-tailed unpaired Student's t-tests, (Mean± SD); **p* < 0.05.
